# Cardiac function and mechanics in systemic sclerosis: a systematic review and meta-analysis

**DOI:** 10.1186/s44156-025-00081-4

**Published:** 2025-07-14

**Authors:** Mihnea Casian, Alina Dima, Ciprian Jurcuț, Laura Andrei, Jamie Edwards, Jamie O’Driscoll, Bogdan A. Popescu, Ruxandra Jurcuț

**Affiliations:** 1 University of Medicine and Pharmacy “Carol Davila”, Str. Dionisie Lupu 37, Sector 2, 020021 Bucharest, Romania; 2 Department of Cardiology, Emergency Institute for Cardiovascular Diseases “Prof. Dr. C. C. Iliescu”, Bucharest, Romania; 3 Cardiovascular Clinical Academic Group St. George’s, University of London and St. George’s University Hospitals NHS Foundation Trust, London, UK; 4 Department of Rheumatology, Colentina Clinical Hospital, Bucharest, Romania; 5 Department of Internal Medicine, “Dr. Carol Davila” Central University Emergency Military Hospital, Bucharest, Romania; 6 Cardiac Investigations, St George’s University Hospitals NHS Foundation Trust, Blackshaw Road, Tooting, London, UK; 7 University of East London, London, UK; 8 University of Leicester, Leicester, UK

**Keywords:** Systemic sclerosis, Subclinical, Cardiac, Echocardiography, TDI, Strain, Speckle-tracking

## Abstract

**Objectives:**

The study aimed to evaluate differences in conventional, tissue Doppler imaging (TDI) and speckle-tracking echocardiographic (STE) parameters of all cardiac chambers between SSc patients and healthy controls.

**Methods:**

A study search strategy based on the Preferred Reporting Items for Systematic Reviews and Meta-Analyses (PRISMA) was performed. MEDLINE, Scopus and Web of Science were searched using the following keywords: “speckle tracking”, “global strain”, “longitudinal strain”, “circumferential strain”, “radial strain”, “atrial strain”, “right ventricular strain”, or “left ventricular strain” and “systemic sclerosis”. Primary pooled analyses were performed on each cardiac parameter independently to determine the weighted mean difference (WMD) between SSc and controls. Further independent subgroup analyses were performed to compare symptomatic vs asymptomatic SSc and diffuse vs limited SSc.

**Results:**

The systematic review and meta-analysis included 41 case–control eligible reports studies with a pooled sample size of 2497 SSc cases and 1439 controls. Significant weighted mean differences (WMD) between SSc patients and healthy controls were identified in septal S’ wave (WMD 0.343 cm/s, CI [− 0.540–0.145], I^2^: 36%, p = 0.001), lateral S’ wave (WMD 0.795 cm/s, CI [− 1.394–0.197], I^2^: 0%, p = 0.009), tricuspid S’ wave (WMD 1.137 cm/s, CI [− 1.784–0.489], I^2^: 84%, p = 0.001), septal e’ wave (WMD 1.398 cm/s, CI [− 2.272–0.523], I^2^: 82%, p = 0.002) and lateral e’ wave (WMD 3.545 cm/s, CI [− 4.990–2.100], I^2^: 71%, p < 0.001) velocities. STE parameters were attenuated in patients with SSc, with impairment of left ventricular global longitudinal (WMD 2.765%, CI [− 3.482–2.049], I^2^: 91%, p < 0.001), circumferential (WMD 3.145%, CI [− 4.181–2.109], I^2^: 79%, p < 0.001), and radial (WMD 4.044%, CI [− 6.199–1.889], I^2^: 0%, p < 0.001) strain, right ventricular free wall (WMD 4.492%, CI [− 6.048–2.937], I^2^: 76%, p < 0.001) and right ventricular global longitudinal strain (WMD 2.843%, CI [− 3.290–2.396], I^2^: 32%, p < 0.001), as well as left (WMD − 8.317%, CI [− 11.873–4.761], I^2^: 82%, p < 0.001) and right (WMD 7.346%, CI [− 10.536–4.156], I^2^: 26%, p < 0.001) atrial reservoir strain.

**Conclusion:**

SSc is associated with significantly impaired cardiac function and mechanics compared to healthy individuals, even in the absence of symptoms or pulmonary hypertension.

**Graphical Abstract:**

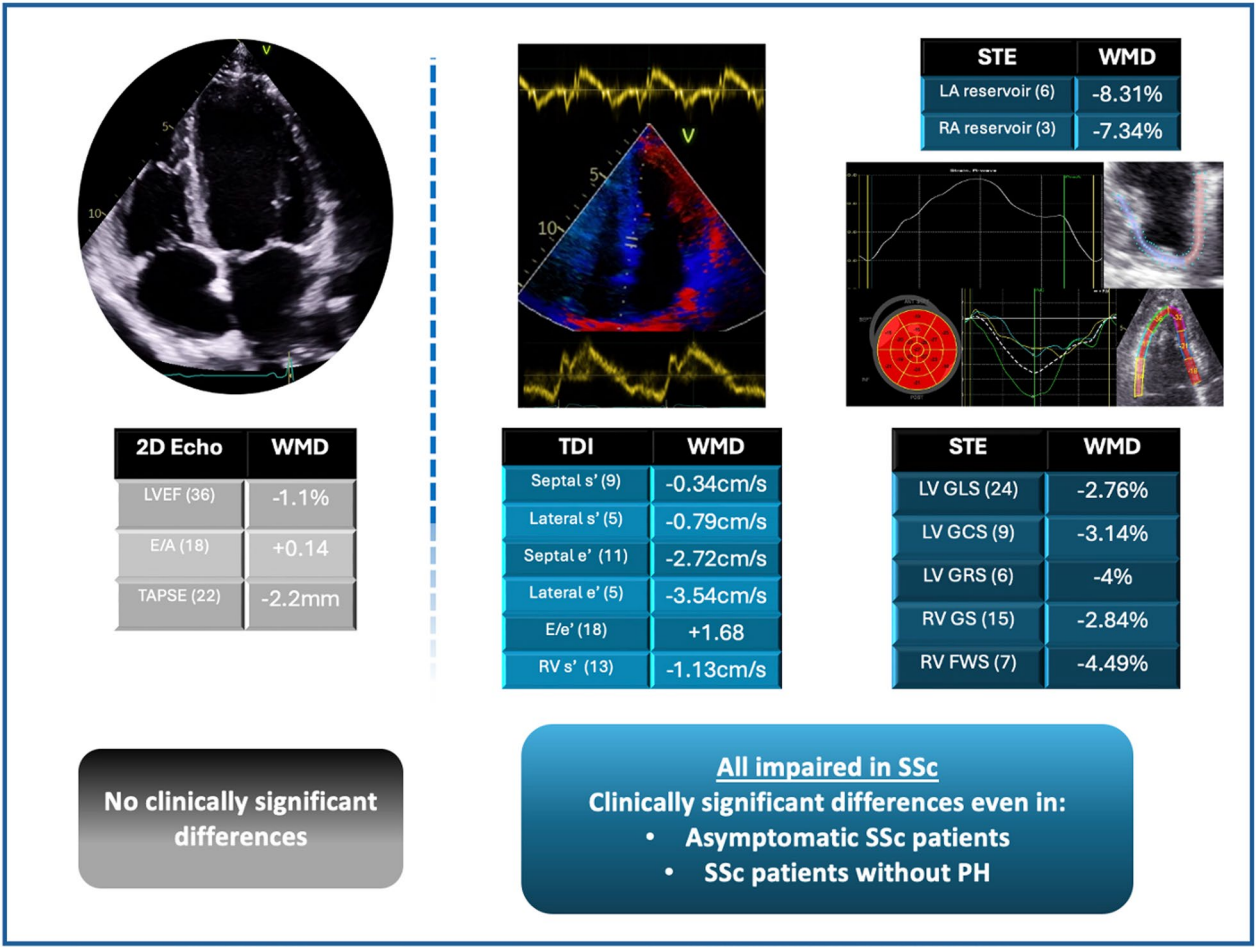

**Supplementary Information:**

The online version contains supplementary material available at 10.1186/s44156-025-00081-4.

## Key messages

- Cardiac involvement in SSc extends beyond pulmonary hypertension, affecting all cardiac chambers, and may be present in asymptomatic patients.
- Our findings support the existence of subclinical myocardial dysfunction in patients with SSc.
- TDI and STE are readily available clinical tools which can identify patients at risk of being affected.

**Supplementary Information:**

The online version contains supplementary material available at 10.1186/s44156-025-00081-4.

## Introduction

Systemic sclerosis (SSc) or scleroderma is a rare connective tissue disease with a complex and far from understood pathophysiology. Vascular insult, tissue fibrosis and complex immune interactions are the hallmarks of the disease, which in time affect multiple organs, including the heart [1]. SSc has the highest case-specific mortality of all the collagen vascular disorders, with prognosis being greatly impacted by the systemic involvement and end-organ damage [1]. Of these, the cardiopulmonary complications are the leading cause of mortality in SSc, with cardiac involvement alone accounting for 36% of deaths [2, 3]. A wide variety of cardiac abnormalities occur in systemic sclerosis, including myocardial fibrosis, epicardial or microvascular coronary artery disease, left ventricular systolic and diastolic dysfunction, pericardial disease, as well supraventricular and ventricular arrhythmias. While pulmonary hypertension (PH) is the better-known and most feared cardiac complication, understanding and detecting the SSc primary heart involvement (SSc-pHI) is the focus of current research efforts [5]. The SSc-pHI is acknowledged as comprising cardiac abnormalities that are predominantly attributable to SSc, rather than other causes and/or complications, which may be subclinical and must be confirmed through diagnostic investigations [5]. Clinically detected cardiac involvement is recognized in only 15–25% of patients, while the rate of detection in asymptomatic patients is dependent on the diagnostic method used [6, 7]. As such, when sensitive tools are used, subclinical cardiac involvement can be proven in 70 to 100% of patients [8–10]. Accurate and early diagnosis of myocardial involvement in patients with SSc is of paramount importance for their prognosis with implications in their clinical management. A normal standard 2D echocardiography may fail to detect abnormalities, at least when compared to other imaging methods, such as cardiac MRI [12]. Thus, the prognostic value of a normal 2D echocardiography is limited [12]. While the cardiac MRI provides valuable incremental prognostic value, it is not widely available and may not be cost-efficient as a first-line imaging method. Tissue Doppler imaging (TDI) and speckle-tracking echocardiography (STE) are emerging as more sensitive and reproducible tools in various clinical scenarios, including systemic autoimmune diseases [13, 14]. Comprehensive data reporting the discrepancies in all TDI and STE parameters between patients with SSc and healthy controls are lacking. A previous similar meta-analysis previously investigated only STE parameters [15].

## Methods

This systematic review aimed to gather the available data regarding TDI and tissue myocardial deformation assessed by STE in patients with SSc compared to healthy controls. Further to this, we were also interested in comparing asymptomatic patients with SSc to healthy as controls, as well patients with diffuse and limited SSc to healthy controls.

The literature search was performed with adherence to the Preferred Reporting Items for Systematic reviews and Meta-Analyses (PRISMA) protocol for reporting on systematic reviews and metanalysis.

To this aim, a protocol for this research was initially developed, detailing the objectives, search strategy and criteria used for study selection, and registered in the international prospective register of systematic reviews, PROSPERO (CRD42022301267) [16].

### Search strategy

Systematic search for relevant articles was conducted in three databases: MEDLINE via PubMed, Scopus and Web of Science Core Collection. In order to identify all available studies which included TDI and/or STE data in patients with SSc, multiple broad search hedges were created specifically for each database, using the following search terms: (“speckle tracking” OR “global strain” OR “longitudinal strain” OR “circumferential strain” OR “radial strain” OR “atrial strain” OR “right ventricular strain” OR “left ventricular strain”) AND “systemic sclerosis”. The search was restricted to records published in English between the 1 st of January 2000 up to and including the 23rd of October 2023. 

### Inclusion criteria

According to the pre-specified protocol, only case–control studies reporting TDI and STE data in patients with SSc, in whom diagnosis was supported by relevant criteria (LeRoy, ACR-EULAR) and with healthy individuals as controls were included [17, 18]. Studies had to report values (means with standard deviation or standard error) of at least one of the following echocardiographic parameters: left ventricular (LV) TDI parameters: septal s’ wave, septal e’ wave, septal a’ wave, lateral wall s’ wave, lateral wall e’ wave, lateral wall a’ wave, LV strain parameters, such as: global longitudinal strain (GLS), circumferential strain (GCS), radial strain (GRS), left atrial (LA) strain parameters: reservoir, conduit, and contractile strain, right ventricular (RV) TDI parameters: free wall s’ wave, RV strain parameters: free wall strain (FWS), global longitudinal strain (RV GLS), or right atrial (RA) strain parameters.

### Exclusion criteria

Studies which were not designed as case–control were excluded. Congress abstracts, short research letters, studies with insufficient data or particularly, case–control studies in which the control population was not made up of healthy individuals, were not included. Studies concerning the paediatric population or animal studies were also excluded. Pulmonary hypertension (PH), coronary heart disease, presence of cardiac related symptoms, or use of immunosuppressive therapies were not used as exclusion criteria, but these aspects were noted for each study, when mentioned by authors.

### Study eligibility

The reference lists of retrieved articles were manually reviewed, and a search for duplicates was conducted after merging the three reference lists. Two reviewers, M.C. and A.D. independently reviewed the titles and available abstracts to judge their relevance for inclusion in analysis. Subsequently, the full text of these potentially eligible reports were fully assessed based on the inclusion and exclusion criteria. Selection results showed a high inter-reader agreement. Discrepancies between the two independent reviewers (M.C. and A.D.) were settled by a third author’s opinion (R.J.). 

### Study quality assessment and data extraction

The risk of bias and methodological quality of the included studies was measured using the Newcastle–Ottawa Quality Assessment Form for Case–Control Studies (NOS) [20]. NOS is a tool designed for the assessment of non-randomized studies, judged on the categories: selection, comparability and exposure; with a maximum achievable score of 9 stars. For the purpose of subgroup analysis, study quality was determined as ‘high’ with a NOS of 8 or 9. Two researchers (M.C. and A.D.) independently scored each of the included papers and resolved any discrepancies via consensus. Using the same standardized spreadsheet, two reviewers (M.C. and A.D.) performed the data extraction from all the eligible reports included.

### Statistical analysis

All statistical analysis was performed using Comprehensive Meta-Analysis (Comprehensive Meta-Analysis Version 3, Biostat, Englewood, NJ, USA). Primary pooled analyses were performed on each cardiac parameter independently to determine the weighted mean difference (WMD) between SSc and controls. Further independent subgroup analyses were performed to compare symptomatic vs asymptomatic SSc and diffuse vs limited SSc. Where possible, meta-regression analyses were also performed to assess the effect of NOS study quality scoring on the relevant parameter. Statistical heterogeneity was tested alongside the pooled primary and subgroup analyses and reported as the I^2^ statistic. A 40% threshold was determined a priori, with heterogeneity above this point considered significant. Fixed effects analysis was preferred unless the I^2^ statistic was beyond this threshold, in which a random-effects analysis was selected as suggested when interstudy variability is confirmed through significant heterogeneity [19]. Furthermore, a post-hoc Egger’s regression test was systematically planned to assess the presence of funnel plot asymmetry with consideration of potential publication bias [20].

## Results

### Literature search

**Fig. 1:**
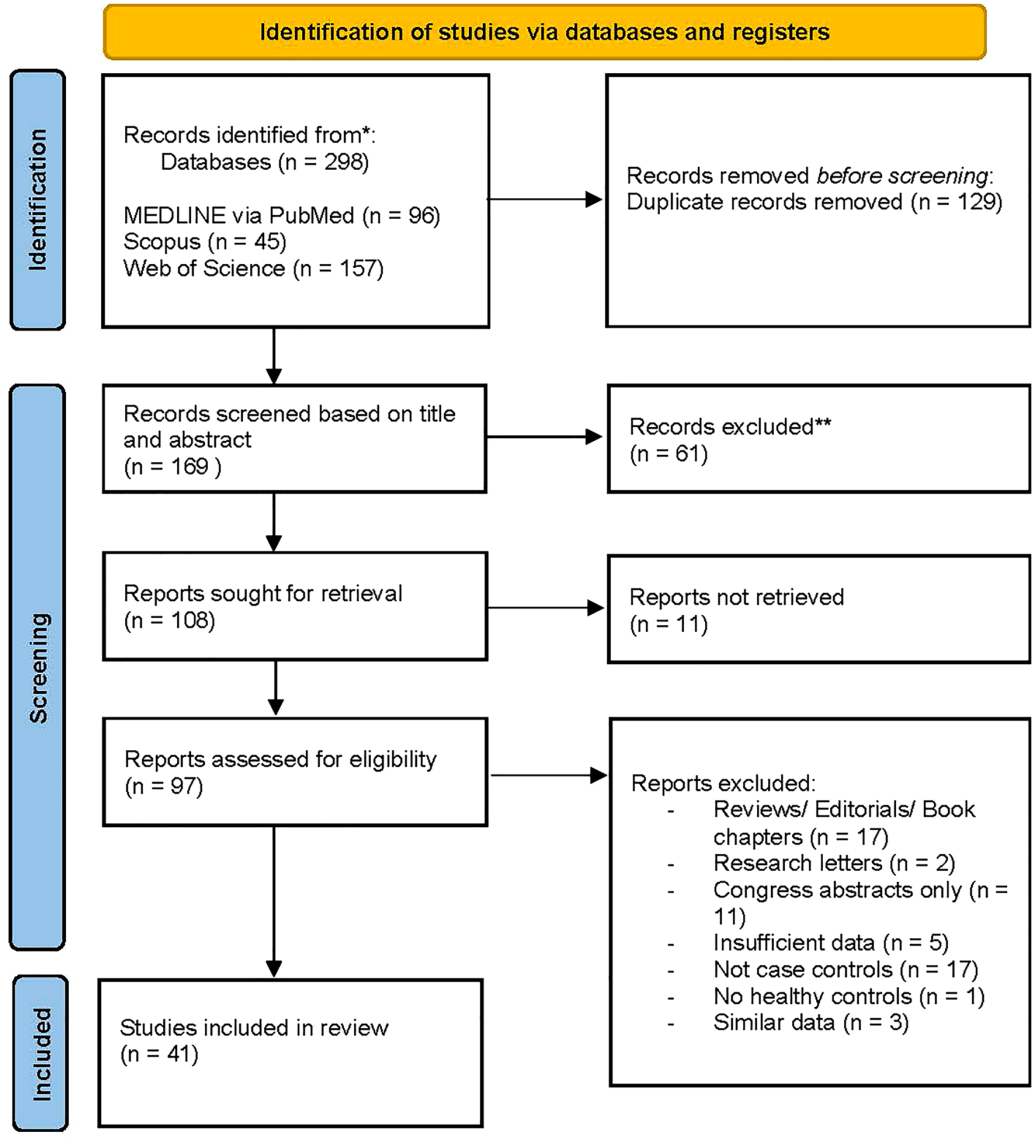
PRISMA flow diagram according to [16]

A total of 298 studies were identified in the three databases using the search terms. The search for duplicates led to exclusion of 129 articles. The remaining 169 articles had their abstracts retrieved and underwent selection using an automated search followed by a manual screen process. 108 articles were sought for retrieval. Out of them, 97 articles were assessed for eligibility. 56 records were excluded after the full-text assessment. One article was excluded because it reported data from patients with SSc and other connective tissue diseases, and the authors were not able to extract the relevant data concerning only patients with SSc [21]. Finally, the results of 41 articles were included in analysis. The PRISMA flow diagram presents the overview of the selection process (see Fig. 1).

Most studies mentioned including patients with limited SSc and diffuse SSc, with one study including only patients with diffuse SSc [22]. PH was confirmed by the authors in 8 studies [23–29], but not all studies mentioned performing right-heart catheterization. Only one study explored the effects of a therapeutic agent (namely Bosentan) on the myocardial function of the SSc patients [31]. Symptoms were inconsistently acknowledged in the articles included. 20 studies included only asymptomatic patients [6, 7, 22, 23, 27, 32–44], while only 3 studies specifically declared enrolling symptomatic patients [25, 28, 29]. Unfortunately, the studies did not consistently acknowledge the presence of traditional cardiovascular risk factors or other comorbidities, which may influence the echocardiographic findings.

Most studies reported TDI and/or STE data for the left and/or right ventricle evaluation, with 6 studies also reporting STE measurements for the left atrium [27, 46–49] and 3 studies for the right atrium [50, 51]. We identified studies which further explored the myocardial function by performing exercise echocardiography [53–55]. Data reporting on multimodality cardiac imaging was found in 3 studies, adding the assessment of myocardial inflammation by PET-CT to TDI and STE data, as well as myocardial tissue characterization through CMR [31, 57, 58]. The 41 studies included in analysis counted for a total number of 2497 SSc cases and 1439 controls. This is merely an estimation, as some studies have been conducted by the same research groups, sharing the respective patient database. In the assessment of potential publication bias, Egger’s regression analyses were run for each pooled analysis, revealing evidence of funnel plot asymmetry for LA conduit strain, LV GRS, E/e’ ratio and RVFWS.

### Echocardiography parameters

#### Left ventricle

A total of 36 studies reported a 2D assessment of the left ventricular systolic function through left ventricular ejection fraction (LVEF) while 24 of them also reported TDI and/or STE parameters. We found that the LVEF was significantly lower in SSc patients when compared to healthy control (WMD 1.123%, CI [− 1.631–0.614], I^2^: 56%, p < 0.001). In addition, two sub-analyses were conducted. There were no significant differences between asymptomatic (WMD 0.721%, CI [− 1.550, 0.108], p = 0.088), with diffuse (WMD 1.536%, CI [− 3.242, 0.170], p = 0.078) or limited SSc patients (WMD 0.097%, CI [-− 1.527, 1.720], p = 0.907) and healthy controls.

**Fig. 2:**
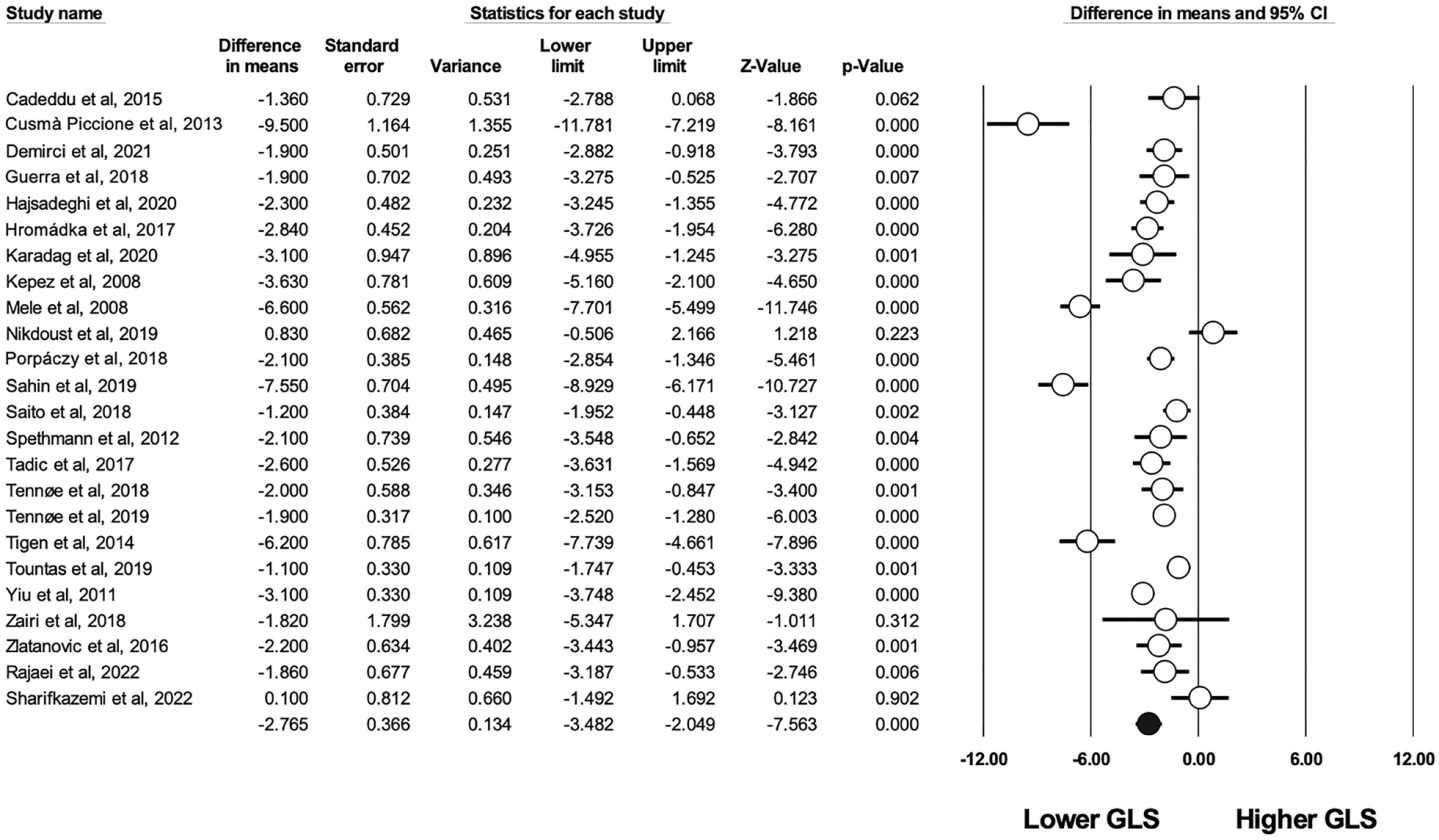
Difference in means in global longitudinal strain (GLS) between patients with SSc and controls

LV GLS was reported in 24 studies (see Fig. 2) [59, 60]. LV GLS was significantly altered in patients with SSc compared to healthy controls (WMD 2.765%, CI [− 3.482–2.049], I^2^: 91%, p < 0.001). The difference remained significant when studies including only asymptomatic patients were analysed (WMD 3.227%, CI [− 4.143–2.311], p < 0.001). The differences were also significant among the two forms of the disease and healthy controls, (diffuse SSc (WMD 4.026%, CI [− 5.840–2.212], p < 0.001), limited (WMD 3.010%, CI [− 4.715–1.306], p = 0.001).

LV GRS values were reported by 6 studies [7, 22, 39, 42, 43, 49]. LV GRS was significantly lower in patients with SSc (WMD 4.044%, CI [− 6.199–1.889], I^2^: 0%, p < 0.001)/LV GCS was mentioned in 9 studies [7, 22, 26, 39, 41, 42, 49, 61]. LV GCS showed significant differences between SSc patients and healthy controls (WMD 3.145%, CI [− 4.181–2.109], I^2^: 79%, p < 0.001). 

Assessment of the LV longitudinal systolic function through TDI parameters using the septal and lateral s’ waves was reported in 9 [6, 22, 24, 32, 36, 37, 42, 47, 53, 62] and 5 studies [6, 23, 24, 32, 47], respectively. Both the septal and lateral s’ wave velocities showed significantly lower values in SSc patients compared to healthy controls (septal s’ wave: WMD 0.343 cm/s, CI [− 0.540–0.145], I^2^: 36%, p = 0.001), lateral s’ wave (WMD 0.795 cm/s, CI [− 1.394–0.197], I^2^: 0%, p = 0.009).

**Fig. 3:**
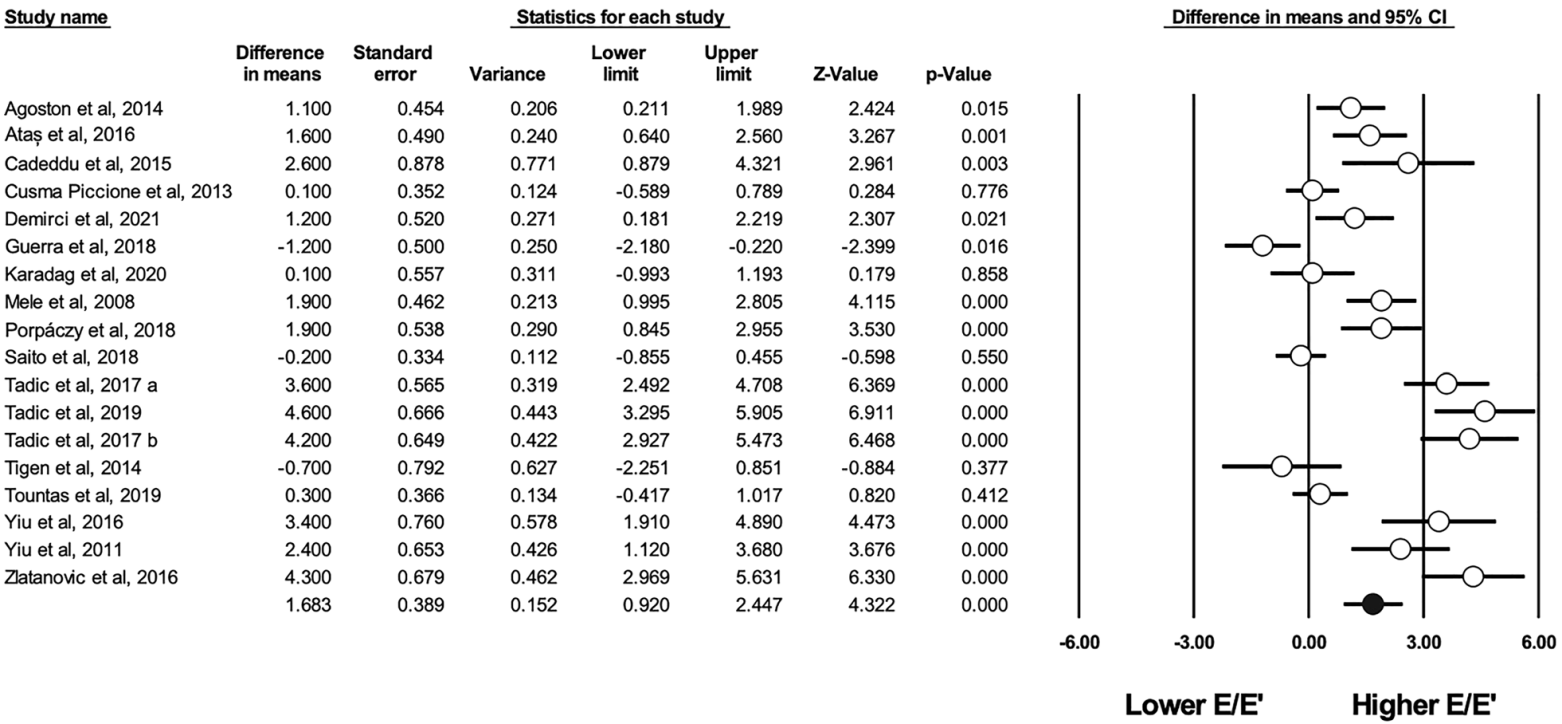
Difference in means in E/e’ ratio between patients with SSc and controls

Diastolic function, assessed using the E/A ratio, was reported in 18 studies. The ratio was significantly lower in patients with systemic sclerosis (WMD 0.140, CI [− 0.201–0.078], I^2^: 67%, p < 0.001). The estimated LV filling pressure (based on the E/e’ ratio) was reported in 18 studies, estimated as significantly higher in affected individuals (WMD 1.683, CI [0.920, 2.447], I^2^: 90%, p < 0.001) (see Fig. 3). Consequently, the septal e’ (WMD 1.398 cm/s, CI [− 2.272–0.523], I^2^: 82%, p = 0.002) and lateral e’ (WMD 3.545 cm/s, CI [− 4.990–2.100], I^2^: 71%, p < 0.001) wave velocities, which were reported in 11 [6, 24, 25, 32, 35, 42, 47, 49, 50, 53, 62] and 5 studies [6, 23, 24, 32, 47, 50], respectively, were significantly lower in affected individuals. The septal and lateral a’ wave velocities were not significantly different between patients with SSc and healthy controls.

#### Left atrium

**Fig. 4:**
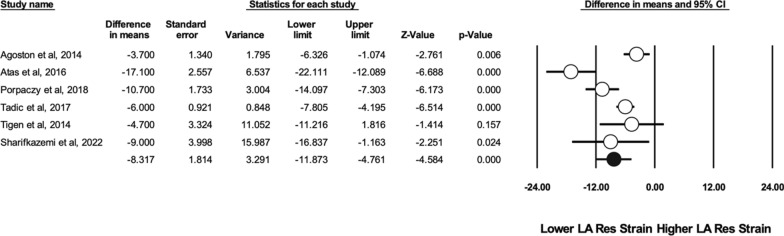
Difference in means in left atrium reservoir strain between patients with SSc and controls

There were 8 studies that reported the indexed left atrial volume (LAVi) [26, 27, 33, 39, 43, 48, 50, 53]. This was found to be significantly higher in individuals with SSc compared to healthy controls (WMD 3.003 ml/m^2^, CI [1.253–4.753], I^2^: 77%, p = 0.001). STE analysis of the left atrium included the reservoir, conduit and contraction strain. The reservoir strain was performed in 6 studies [27, 46–49] and showed a significant impairment in patients with SSc (WMD 8.317%, CI [− 11.873–4.761], I^2^: 82%, p < 0.001) (see Fig. 4). A significant difference was also seen in the conduit strain (WMD 2.875%, CI [− 4.947–0.804], I^2^: 53%, p = 0.007), which was performed in 4 studies [46–48]. The contraction strain was performed in 4 studies, without any significant differences being found [27, 46, 47].

#### Right ventricle

A total of 22 studies included TDI and/or STE data concerning RV systolic function. TAPSE was reported in 22 studies, out of which 6 mentioned including patients with PH [23, 24, 28, 29]. TAPSE was significantly reduced in individuals with SSc compared to healthy controls (WMD 2.299 mm, CI [− 3.382–1.216], I^2^: 93%, p < 0.001). The difference remained significant when only patients without PH were considered (WMD 2.428 mm, CI [− 3.960–0.896], p = 0.002).

RV FAC was reported by 13 studies and found to be significantly reduced in the SSc patients (WMD 3.317%, CI [− 5.179–0.772], I^2^: 80%, p = 0.002). However, the difference lost its significance when RV FAC was compared exclusively between asymptomatic SSc patients and healthy controls (WMD 2.548%, CI [− 6.750–1.654], p = 0.235) [22, 33, 38, 40, 41, 44]. RVEF assessed by 3D echocardiography was performed in only 2 studies, which did not include patients with PAH [38, 40]. The RVEF was significantly lower in affected individuals (WMD 10.491%, CI [− 13.431–7.551], I^2^: 64%, p < 0.001).

The right ventricular free wall s’ wave was reported by 13 studies, 3 of them also including patients with PH [23, 25, 26]. Patients with SSc had significantly lower s’ velocities compared to healthy controls (WMD 1.137 cm/s, CI [− 1.784–0.489], I^2^: 84%, p = 0.001). The difference remained significant even after studies including patients with PH were excluded (WMD 1.280 cm/s, CI [− 2.135–0.426], p = 0.003).

**Fig. 5:**
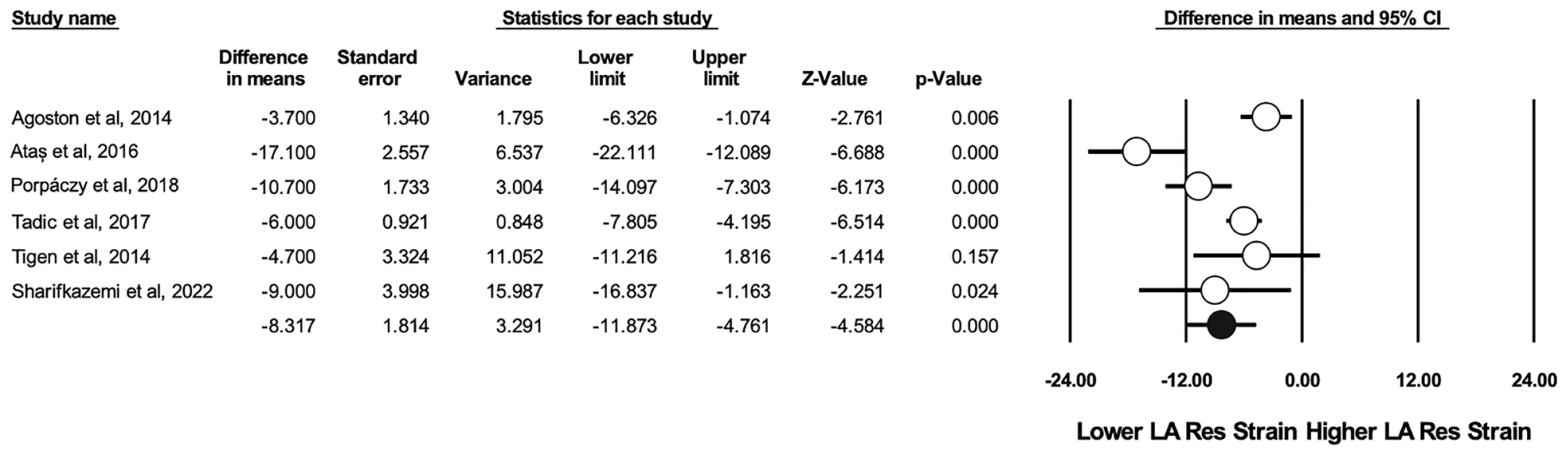
Difference in means in right ventricular free wall strain (RV FWS) between patients with SSc and controls

RVFWS was reported in 7 studies, with values being significantly more impaired in individuals with SSc (WMD 4.492%, CI [− 6.048–2.937], I^2^: 76%, p < 0.001) (see Fig. 5). One study also included patients with PH [30]. Its exclusion did not alter the difference (WMD 4.647%, CI [− 6.488–2.806], p < 0.001). More studies [15] reported RV GS, which was found to be significantly attenuated in patients with SSc (WMD 2.843%, CI [− 3.290–2.396], I^2^: 32%, p < 0.001). Only one of the studies also included patients with PH [25]. However, after its exclusion from the analysis, the difference remained significant (WMD 2.810%, CI [− 3.277–2.344], p < 0.001).

#### Right atrium

STE analysis of the right atrium was performed in 3 studies, which did not include patients with PH [50, 51]. Right atrial reservoir strain was found to be significantly more impaired in individuals with SSc compared to healthy controls (WMD 7.346%, CI [− 10.536–4.156], p < 0.001). Right atrial conduit (WMD 5.396%, CI [− 9.098–1.693], p = 0.004) and contraction (WMD 3.216%, CI [− 4.754–1.679], p < 0.01) strain were performed in only two studies [51, 52] and showed significant differences. 

## Discussion

In the present systematic review and meta-analysis, we investigated TDI and STE derived parameters of cardiac mechanics in patients diagnosed with SSc compared to healthy controls. To the best of our knowledge, this is the most comprehensive meta-analysis addressing this subject. Previous meta-analyses investigating these parameters in other systemic autoimmune disorders, such as SLE and RA, as well as in SSc have been published previously [14, 15, 63]. Specifically, the meta-analysis concerning patients with SSc included less studies, 31 compared to 41 in the present study, and addressed only STE parameters, without reporting TDI parameters [15]. These studies highlighted that individuals with systemic autoimmune disorders show impaired left ventricular longitudinal systolic function compared to healthy controls, quantified by GLS. Notably, impaired myocardial deformation has been confirmed in all four cardiac chambers in SSc, as the majority of STE parameters showed lower values compared to healthy controls [15]. Our study also demonstrated that LV GLS is significantly impaired in patients with SSc. Interestingly, our analysis showed a significant difference in LVEF between patients and controls, despite most studies showing similar LVEF values. However, a 1.1% difference in LVEF, although statistically significant, does not hold clinical relevance. LVEF assessed through Simpson’s bi-plane method is influenced by interobserver variability, which can vary by up to 10%. This is improved by 3D measured LVEF, however it was not routinely performed [64]. GLS reproducibility was shown to be superior to that of conventional echocardiographic measures [63, 65, 66]. The WMD in LV GLS was greater and far more clinically significant in our analysis (2.765%) compared to the WMD of 1.1% in LVEF. It has been established previously that a reduction with just 1 unit in LV GLS is associated with a 12% increased risk of major adverse cardiac events in an otherwise low-risk general population [68]. Moreover, the difference maintained its significance when asymptomatic SSc patients were compared with healthy controls, a comparison not explored in the previous meta-analysis [15]. This highlights the importance of LV GLS in identifying individuals with SSc at risk of having subclinical myocardial involvement, which would be otherwise undiagnosed if only traditional echocardiographic parameters, such as LVEF, were to be used. LV systolic dysfunction quantified by LVEF has been identified only in a minority of patients (5.2%) [69]. By contrast, myocardial abnormalities were detected in all patients by nuclear techniques and in 75% of patients when using cardiac MRI [70, 71]. Furthermore, the difference in GLS between affected individuals and healthy controls appears to be slightly greater in patients with a diffuse SSc than in those with a limited cutaneous SSc phenotype (4.0% versus 3.0%). This is in line with the higher rates of cardiac involvement reported in diffuse SSc, as the anti-Scl70 antibodies, the immune hallmark for this more clinically severe SSc phenotype, are associated with more severe systemic organ damage [72, 73]. Thus, GLS is a valuable and readily available tool, addressing an unmet clinical need. It should be systematically performed in patients with SSc, regardless of the presence of symptoms, in order to identify patients who might benefit from further investigations, close monitoring or perhaps even treatment escalation.

LV GRS and GCS are more frequently used in research settings, whereas GLS is more accessible for day-to-day clinical use. In addition to the longitudinal impairment, patients with SSc also have attenuated radial and circumferential strain, suggesting all three LV contractile components are affected in a similar manner, potentially by fibrosis and/or inflammation. Myocardial tissue abnormalities, such as late-gadolinium enhancement (LGE) and edema can be identified in up to 26.4% of asymptomatic patients [75]. These findings are relevant, as there is a direct correlation between LGE and the burden of ventricular arrhythmias, which has shown prognostic significance in the Genetics versus Environment in Scleroderma Outcome Study (GENISOS) [75, 76]. Patients with ventricular arrhythmias had a 2.18-fold increase in the risk of sudden cardiac death compared to arrhythmia-free patients [76].

Detecting diastolic dysfunction is important in patients with SSc, as it is a recognized mortality predictor, exceeding PH [28]. Disease-related pathogenic mechanisms, such as myocardial fibrosis, increased serum levels of tissue inhibitor matrix metalloproteinase and impaired endothelial function, contribute to the impairment of diastolic function, which explains the greater prevalence of diastolic dysfunction in patients with SSc compared to healthy individuals [74, 77]. As expected, we confirmed that the E/A ratio is significantly lower in patients with SSc, albeit the 0.14 difference might be only statistically significant and not clinically relevant. Tissue Doppler velocities are important for the assessment of LV systolic longitudinal function and diastolic function. We confirmed statistically significant differences in all TDI parameters (E/e’ ratio, septal and lateral s’ and e’ wave velocities), except for the septal and lateral a’ waves. The WMD of 1.683 for the E/e’ ratio can be clinically significant and bears prognostic significance in certain populations. For instance, a 1 unit increase in the ratio has been associated with an increased risk of mortality in most studies in patients with heart failure and preserved ejection fraction [78]. The greatest difference was noted in the lateral e’ wave velocities (3.545 cm/s). Some risk factors for low e’ velocities in this setting were reported to be disease duration, age and arterial hypertension [79]. Importantly, e’ wave velocities have prognostic implications, as each standard deviation decrease augments mortality rates 3.2 times [79]. Adding evidence to the greater prevalence of diastolic dysfunction, patients with SSc had significantly greater LAVi compared to healthy individuals.

Left atrial reservoir strain has become clinically relevant and can be used in estimating LV filling pressures, particularly in patients with equivocal findings [80]. Values less than 18% predicted elevated LV filling pressures better than LA volume and conventional Doppler parameters in patients with preserved LVEF [81]. Left atrial reservoir strain values were lower in patients with SSc compared to healthy controls (WMD 8.317%). It should be noted that the difference seems more noteworthy compared to other parameters, such as LAVi (WMD 3 ml/m^2^), E/A ratio (WMD 0.14) or E/e’ ratio (WMD 1.683), prompting clinicians to routinely perform this parameter in patients with SSc. Left atrial reservoir strain offers incremental value in cardiovascular risk stratification and long-term prognosis in several distinct populations, including community-based cohorts, patients with valvular disease or cardiomyopathies, with several thresholds having been proposed in each situation [82–84]. In particular, left atrial function may demonstrate alterations prior to changes in the left atrial volume [86].

As with the left-side cardiac chambers, the right ventricle and atrium may also be affected, even in the absence of PH. Right ventricular involvement is present even in the early stages of the disease in the absence of PH [62]. Early RV dysfunction is strongly associated with the presence of anti-Scl70 antibodies and extent of cutaneous and pulmonary involvement [23, 32]. Moreover, this occurs independently of the systolic pulmonary artery pressures or pulmonary function, suggesting that primary RV myocardial involvement is a distinct entity, not just a consequence of pressure overload [11]. Our analysis also confirms this aspect, as excluding patients with PH did not alter the significance of the difference seen in the velocity of the tricuspid S’ wave, RVFWS or RVGS. The clinical significance of these findings has not been established outside the context of PH, tricuspid regurgitation or heart failure. RVGS seemed more popular than RVFWS, as more studies chose to report it. However, RVFWS may hold superior prognostic value compared to RVGS, which has been demonstrated in patients with heart failure and PH [87, 88]. Adding further evidence to the concept of primary right heart involvement, all the right atrial strain indices were impaired in patients with SSc without PH, with the reservoir strain demonstrating the greatest WMD. Right atrial dysfunction is a predictor for mortality and hospitalization in patients with pulmonary arterial hypertension independent of right atrial size, however its clinical implications in patients without PH are not established [89].

The role of these parameters in the clinical management of patients with SSc is yet to be defined, apart from identifying higher-risk patients given their established prognostic value [90]. However, it seems clear that patients found to have abnormal cardiac mechanics should be followed up closely and perhaps referred for further investigations. These may include the assessment of ventricular arrhythmias with Holter ECG monitors, as well as other imaging techniques, such as cardiac MRI for myocardial tissue characterization. The benefit of commencing cardioprotective drugs based solely on STE parameters is yet to be determined.

### Study limitations

Heterogeneity was high considering the variability between studies included in the analysis, which was expected. Some of the studies reported data from a small number of patients, resulting in a low number of effect sizes included within the analysis, which would explain the wider confidence intervals of some results. Larger scale studies which are adequately powered to detect differences in some unconventional echocardiographic parameters, such as RA and LA strain, will allow for more robustly powered pooled analysis in the future. Additionally, there was evidence of funnel plot asymmetry for pooled analyses of LA conduit strain, LV GRS, E/e’ ratio and RVFWS, suggesting publication bias. Publication bias remains a potential and inherent problem with secondary pooled analyses and thus should be appropriately considered in the interpretation of the changes in for these parameters. Some studies did not specifically disclose PH and/or symptoms as part of their inclusion or exclusion criteria. While several specifically mentioned adhering to the relevant protocols and/or guidelines regarding image acquisition, it remains uncertain whether all studies reporting RV parameters relied on RV-focused views or standard four-chamber views. This of course may significantly impact the values reported for RV and RA parameters. Another concern stems from the strain imaging software used from different vendors. Studies reported using GE, Philips or Toshiba software, however not all studies disclosed this aspect in their protocol. Therefore, the impact of a potential machine-related variability on the current findings cannot be excluded. A previous inter-vendor study showed a small, yet statistically significant variation among vendors [67].

## Conclusion

SSc is associated with significantly impaired cardiac mechanics in all four chambers compared to healthy individuals. These findings support the existence of myocardial dysfunction in SSc, which may occur in either forms of the disease, even in asymptomatic patients. Importantly, right atrial and ventricular impairment occurs even in the absence of PH. This is further evidence that they should be measured systematically and routinely in patients with SSc, regardless of symptoms or PH, as they hold prognostic significance and identify higher-risk patients.

## Supplementary Information

Supplementary Material 1.

Supplementary Material 2.

Supplementary Material 3.

Supplementary Material 4.

Supplementary Material 5.

## Data Availability

Data is provided in the supplementary files.
