# Supplementary material for: Cardiac function and mechanics in systemic sclerosis: a systematic review and meta-analysis
Source: Echo Res Pract. 2025 Jul 14;12:18. doi: 10.1186/s44156-025-00081-4 (PMC12257727; doi:10.1186/s44156-025-00081-4)
Supplement: Supplementary file 1 — Supplementary Material 1. [file 44156_2025_81_MOESM1_ESM.pdf]

Articles

	ARTICLE	AUTHORS	Year	Country	Case	Female (%)	Age (years)	Controls	Echo software	Symptoms	PH
1	Left atrial dysfunction detected by speckle tracking in patients with systemic sclerosis	G. Agoston, L. Gargani, M. H. Miglioranza, M. Caputo, L. P. Badano, A. Moreo, D. Muraru, S. Mondillo, A. M. Pignone, M. M. Cerinic, R. Sicari, E. Picano and A. Varga	2014	Italy	42	95	50±14	42	GE	N/A	0
2	Evaluation of left atrial volume and function in systemic sclerosis patients using speckle tracking and real-time three-dimensional echocardiography	H. Ataş, A. Kepez, K. Tigen, F. Samadov, G. Özen, A. Cincin, M. Sünbül, M. Bozbay, H. Direşkeneli and Y. Başaran	2016	Turkey	41	92.7	49.5±10.6	38	Philips	N/A	0
3	Contractile reserve in systemic sclerosis patients as a major predictor of global cardiac impairment and exercise tolerance	C. Cadeddu, M. Deidda, G. Giau, M. Lilliu, F. Cadeddu, G. Binaghi, M. N. Mura, M. Farci, S. Del Giacco, P. E. Manconi and G. Mercuro	2015	Italy	45	91.1	60.4±10.3	20	Toshiba	N/A	N/A
4	Role of 2D strain in the early identification of left ventricular dysfunction and in the risk stratification of systemic sclerosis patients	M. Cusmà Piccione, C. Zito, G. Bagnato, G. Oreto, G. Di Bella, G. Bagnato and S. Carerj	2013	Italy	29	96.5	65±4	30	My-lab	0	0
5	Myocardial and vascular dysfunction in systemic sclerosis: the potential role of noninvasive assessment in asymptomatic patients	A. D'Andrea, P. Caso, S. Cuomo, F. Scotto di Uccio, R. Scarafile, G. Salerno, S. Romano, S. Stisi, M. Scherillo and R. Calabrò	2007	Italy	33	N/A	N/A	30	N/A	0	0
6	Right atrial morphology and function in patients with systemic sclerosis compared to healthy controls: a two-dimensional strain study	A. D'Andrea, M. D'Alto, M. Di Maio, S. Vettori, N. Benjamin, R. Cocchia, P. Argiento, E. Romeo, G. Di Marco, M. G. Russo, G. Valentini, R. Calabrò, E. Bossone and E. Grünig	2016	Italy	90	75	52.4±15.2	55	GE	N/A	0
7	Early impairment of myocardial function in systemic sclerosis: non-invasive assessment by Doppler myocardial and strain rate imaging	A. D'Andrea, S. Stisi, S. Bellissimo, F. Vigorito, F. Scotto di Uccio, N. Tozzi, F. Moscato, E. Pezzullo, R. Calabrò and M. Scherillo	2005	Italy	23	N/A	56.3±8.2	25	GE	0	1
8	Associations between left ventricular myocardial involvement and endothelial dysfunction in systemic sclerosis: noninvasive assessment in asymptomatic patients	A. D'Andrea, S. Stisi, P. Caso, F. S. Uccio, S. Bellissimo, G. Salerno, R. Scarafile, L. Riegler, S. Cuomo, R. Citro, M. Scherillo and R. Calabrò	2007	Italy	33	N/A	56.3±8.2	30	GE	0	1
9	The evaluation of right ventricle dyssynchrony by speckle tracking echocardiography in systemic sclerosis patients	M. Demirci, B. Ozben, M. Sunbul, A. Cincin, Y. E. Gurel, T. Bayram Guctekin, Z. Dogan, Y. Sahinkaya, H. Direskeneli, K. Tigen and N. Sayar	2021	Turkey	55	92.3	50.5±11.3	45	Philips	1	1
10	Right ventricular and atrial functions in systemic sclerosis patients without pulmonary hypertension. Speckle-tracking echocardiographic study	E. Durmus, M. Sunbul, K. Tigen, T. Kivrak, G. Ozen, I. Sari, H. Direskeneli and Y. Basaran	2015	Turkey	40	N/A	48.5±11.4	40	GE	N/A	0
11	Global longitudinal strain measured by speckle tracking identifies subclinical heart involvement in patients with systemic sclerosis	F. Guerra, G. Stronati, C. Fischietti, A. Ferrarini, L. Zuliani, G. Pomponio, A. Capucci, M. G. Danieli and A. Gabrielli	2018	Italy	52	88.5	54.6±16.1	52	GE	0	0
12	The relationship between global longitudinal strain and pulmonary function tests in patients with scleroderma and normal ejection fraction and pulmonary artery pressure: a case-control study	S. Hajsadeghi, S. Mirshafiee, M. Pazoki, V. Moradians, P. Mansouri, N. Kianmehr and A. Iranpour	2020	Iran	30	80	45.9±11.7	30	Philips	N/A	0
13	Myocardial fibrosis detected by magnetic resonance in systemic sclerosis patients - Relationship with biochemical and echocardiography parameters	M. Hromádka, J. Seidlerová, D. Suchý, D. Rajdl, J. Lhotský, J. Ludvík, R. Rokyta and J. Baxa	2017	Czech Republic	33	88	55.5±12.5	20	GE	0	0
14	Evaluation of left and right ventricle by two-dimensional speckle tracking echocardiography in systemic sclerosis patients without overt cardiac disease	D. T. Karadag, T. Sahin, S. Tekeoglu, O. O. Işik, A. Yazici, F. C. Eraldemir and A. Cefle	2020	Turkey	47	89.4	52.1±12.4	36	GE	0	0
15	Detection of subclinical cardiac involvement in systemic sclerosis by echocardiographic strain imaging	A. Kepez, A. Akdogan, L. E. Sade, A. Deniz, U. Kalyoncu, O. Karadag, M. Hayran, K. Aytemir, I. Ertenli, S. Kiraz, M. Calguneri, G. Kabakci and L. Tokgozoglu	2008	Turkey	27	96	46±10	27	GE	0	0
16	Speckle-tracking-derived strain and strain-rate analysis: A technique for the evaluation of early alterations in right ventricle systolic function in patients with systemic sclerosis and normal pulmonary artery pressure	C. Matias, L. P. D. Isla, M. Vasconcelos, C. Almería, J. L. Rodrigo, V. Serra and J. Zamorano	2009	Spain	17	94	56 (43.8-71.5)	22	Philips	0	0
17	Abnormalities of left ventricular function in asymptomatic patients with systemic sclerosis using Doppler measures of myocardial strain	D. Mele, S. Censi, R. La Corte, E. Merli, A. Lo Monaco, A. Locaputo, C. Ceconi, F. Trotta and R. Ferrari	2008	Italy	35	97	55±13	35	GE	0	0
18	Essential Hypertension Worsens Left Ventricular Contractility in Systemic Sclerosis	V. Mercurio, A. M. Hinze, L. K. Hummers, F. M. Wigley, A. A. Shah and M. Mukherjee	2021	USA	138	87.7	54.3±12.6	80	Philips	N/A	0
19	Unique Abnormalities in Right Ventricular Longitudinal Strain in Systemic Sclerosis Patients	M. Mukherjee, S. E. Chung, V. K. Ton, R. J. Tedford, L. K. Hummers, F. M. Wigley, T. P. Abraham and A. A. Shah	2016	USA	138	87.7	54.3±12.6	40	Philips	N/A	0
20	Left and right ventricular functional status in patients suffering from scleroderma with normal pulmonary arterial pressure	F. Nikdoust, Z. Tahmasebi, A. Mostafavi and S. A. H. Tabatabaei	2019	Iran	35	N/A	N/A	35	N/A	N/A	0
21	Relation of Right Atrial Mechanics to Functional Capacity in Patients With Systemic Sclerosis	Á. Nógrádi, A. Porpáczy, L. Porcsa, T. Minier, L. Czirják, A. Komócsi and R. Faludi	2018	Hungary	70	90	57±12	25	Philips	N/A	0
22	Evaluation of right ventricular function performed by 3D-echocardiography in scleroderma patients	E. Pigatto, D. Peluso, E. Zanatta, P. Polito, P. Miatton, K. Bourji, L. P. Badano, L. Punzi and F. Cozzi	2015	Italy	45	93	N/A	43	GE	0	0
23	Impairment of Left Atrial Mechanics Is an Early Sign of Myocardial Involvement in Systemic Sclerosis	A. Porpáczy, Á. Nógrádi, D. Kehl, M. Strenner, T. Minier, L. Czirják, A. Komócsi and R. Faludi	2018	Hungary	72	92	57.1±11	30	Philips	N/A	0
24	Subclinical biventricular systolic dysfunction in patients with systemic sclerosis	T. Şahin Ş, N. Yılmaz, B. Cengiz, S. Yurdakul, Y. Çağatay, E. Kaya, S. Aytekin and Ş. Yavuz	2019	Turkey	47	91.4	48.2±12.4	20	Philips	N/A	0
25	Mechanics and prognostic value of left and right ventricular dysfunction in patients with systemic sclerosis	M. Saito, L. Wright, K. Negishi, N. Dwyer and T. H. Marwick	2018	Australia	103	79	63 (57-71)	103	GE	N/A	1
26	Early right ventricular systolic dysfunction in patients with systemic sclerosis without pulmonary hypertension: a Doppler Tissue and Speckle Tracking echocardiography study	S. Schattke, F. Knebel, A. Grohmann, H. Dreger, F. Kmezik, G. Riemekasten, G. Baumann and A. C. Borges	2010	Germany	22	77.3	57±13.4	22	GE	N/A	0
27	Two-dimensional speckle tracking of the left ventricle in patients with systemic sclerosis for an early detection of myocardial involvement	S. Spethmann, H. Dreger, S. Schattke, G. Riemekasten, A. C. Borges, G. Baumann and F. Knebel	2012	Germany	22	77.3	57.1±13.3	22	GE	0	0
28	The relationship between left ventricular deformation and heart rate variability in patients with systemic sclerosis: Two- and three-dimensional strain analysis	M. Tadic, M. Zlatanovic, C. Cuspidi, B. Ivanovic, A. Stevanovic, N. Damjanov, V. Kocijancic and V. Celic	2017	Serbia	49	92	54±10	38	GE	0	0
29	Systemic sclerosis impacts right heart and cardiac autonomic nervous system	M. Tadic, M. Zlatanovic, C. Cuspidi, A. Stevanovic, V. Celic, N. Damjanov and V. Kocijancic	2018	Serbia	45	94	55±10	35	GE	0	0
30	Left atrial phasic function and heart rate variability in patients with systemic sclerosis: A new part of the old puzzle	M. Tadic, M. Zlatanovic, C. Cuspidi, A. Stevanovic, V. Kocijancic, S. U. Pavlovic, N. Damjanov and V. Celic	2017	Serbia	44	97	53±10	33	GE	0	1
31	Left Ventricular Diastolic Dysfunction Predicts Mortality in Patients With Systemic Sclerosis	A. H. Tennoe, K. Murbraech, J. C. Andreassen, H. Fretheim, T. Garen, E. Gude, A. Andreassen, S. Aakhus, O. Molberg and A. M. Hoffmann-Vold	2018	Norway	249	83	58±14	65	GE	1	1
32	Systolic Dysfunction in Systemic Sclerosis: Prevalence and Prognostic Implications	A. H. Tennøe, K. Murbræch, J. C. Andreassen, H. Fretheim, Ø. Midtvedt, T. Garen, H. Dalen, E. Gude, A. Andreassen, S. Aakhus, Ø. Molberg and A. M. Hoffmann-Vold	2019	Norway	277	82	57±13	65	GE	1	1
33	Regional myocardial dysfunction assessed by two-dimensional speckle tracking echocardiography in systemic sclerosis patients with fragmented QRS complexes	K. Tigen, M. Sunbul, G. Ozen, E. Durmus, T. Kivrak, A. Cincin, B. Ozben, H. Atas, H. Direskeneli and Y. Basaran	2014	Turkey	53	88.7	49.1±11	26	GE	N/A	N/A
34	Mechanics of early ventricular impairment in systemic sclerosis and the effects of peripheral arterial haemodynamics	C. Tountas, A. D. Protogerou, V. K. Bournia, S. Panopoulos, G. Konstantonis, M. G. Tektonidou, A. Gournizakis, D. Beldekos and P. P. Sfikakis	2019	Greece	95	88	53.3±13.7	54	GE	0	0
35	Impact of pulmonary fibrosis and elevated pulmonary pressures on right ventricular function in patients with systemic sclerosis	K. H. Yiu, M. K. Ninaber, L. J. Kroft, A. A. Schouffoer, J. Stolk, H. U. Scherer, J. Meijs, J. de Vries-Bouwstra, H. F. Tse, V. Delgado, J. J. Bax, T. W. Huizinga and N. A. Marsan	2016	Netherlands	102	62	54±14	36	GE	N/A	1
36	Left ventricular dysfunction assessed by speckle-tracking strain analysis in patients with systemic sclerosis: relationship to functional capacity and ventricular arrhythmias	K. H. Yiu, A. A. Schouffoer, N. A. Marsan, M. K. Ninaber, J. Stolk, T. V. Vlieland, R. W. Scherptong, V. Delgado, E. R. Holman, H. F. Tse, T. W. Huizinga, J. J. Bax and A. J. Schuerwegh	2011	Netherlands	104	77	54±12	34	GE	0	0
37	Speckle tracking echocardiography in systemic sclerosis: A useful method for detection of myocardial involvement	I. Zairi, K. Mzoughi, Z. Jnifene, S. Kamoun, M. Jabeur, F. Ben Moussa and S. Kraiem	2019	Tunisia	25	96	53.6±13.4	25	GE	N/A	N/A
38	Cardiac mechanics and heart rate variability in patients with systemic sclerosis: the association that we should not miss	M. Zlatanovic, M. Tadic, V. Celic, B. Ivanovic, A. Stevanovic and N. Damjanov	2017	Serbia	41	93	56±11	30	GE	0	0
39	Ventricular and atrial function assessment with transthoracic echocardiography in patients with rheumatic inflammatory disease	S. Norouzi, A. Khalaji, M. Namazi, S. S. Rezaei, A. H. Behnoush and M. Masoumi	2022	Iran	21	90.4	41.3±13.7	64	N/A	0	0
40	Evaluation of the Left Ventricular Function in Patients with Scleroderma with Normal Pulmonary Artery Pressure Using Myocardial Strain Analysis: A Cross-Sectional Study	E. Rajaei, N. P. Isfahani, N. Akiash, M. Mohammadi and K. Mowla	2022	Iran	30	83.2	41±8.9	30	N/A	0	0
41	Diagnosis of Simultaneous Atrial and Ventricular Mechanical Performance in Patients with Systemic Sclerosis	M. Sharifkazemi, M. Nazarinia, A. Arjangzade, M. Goldust and Z. Hooshanginezhad	2022	Iran	37	N/A	45.7±115	37	Siemens	N/A	0
