# Supplementary material for: Cardiac function and mechanics in systemic sclerosis: a systematic review and meta-analysis
Source: Echo Res Pract. 2025 Jul 14;12:18. doi: 10.1186/s44156-025-00081-4 (PMC12257727; doi:10.1186/s44156-025-00081-4)

Funnel Plot Asymmetry for LA Conduit Strain

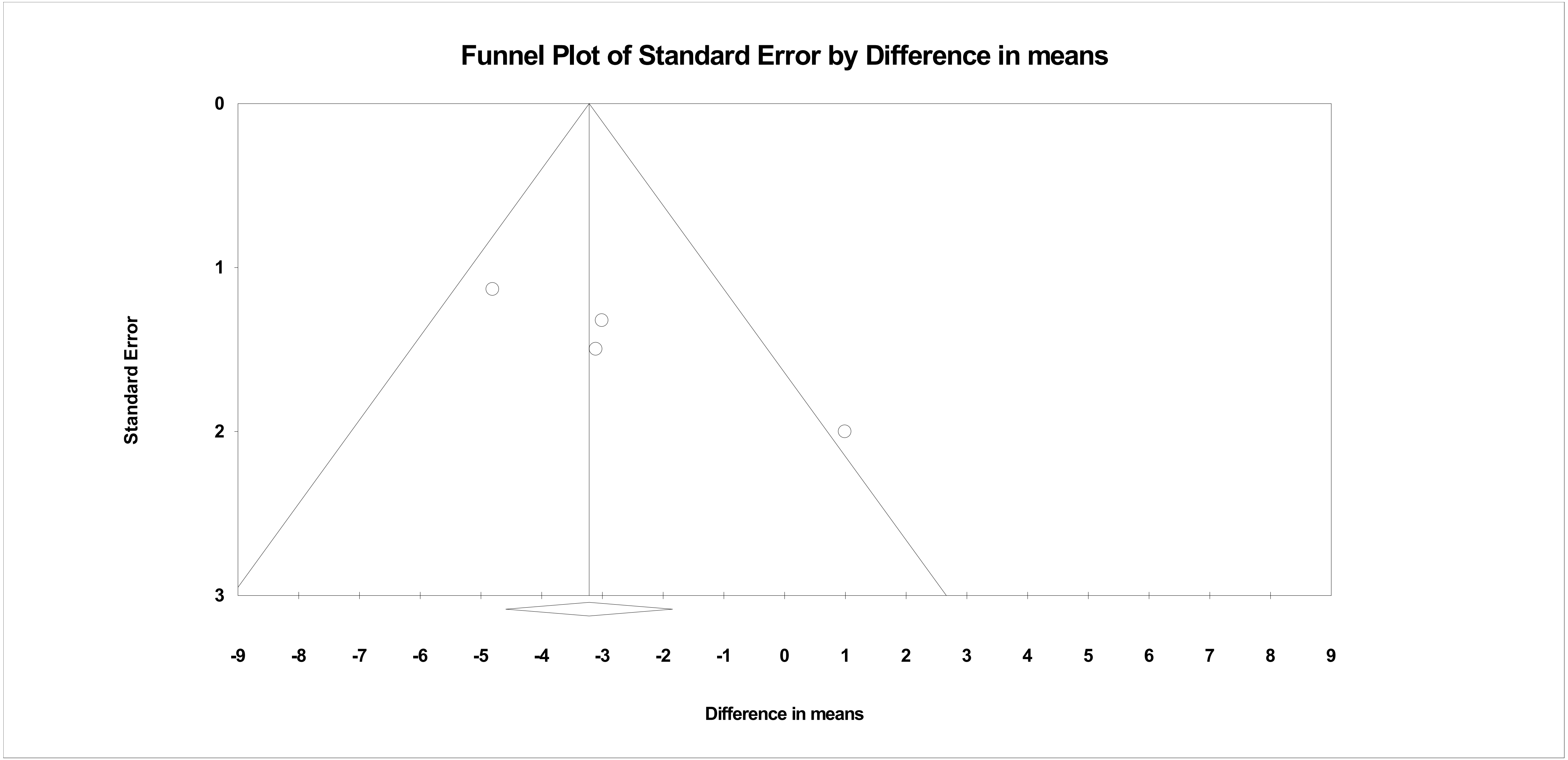

Funnel Plot Asymmetry for GRS

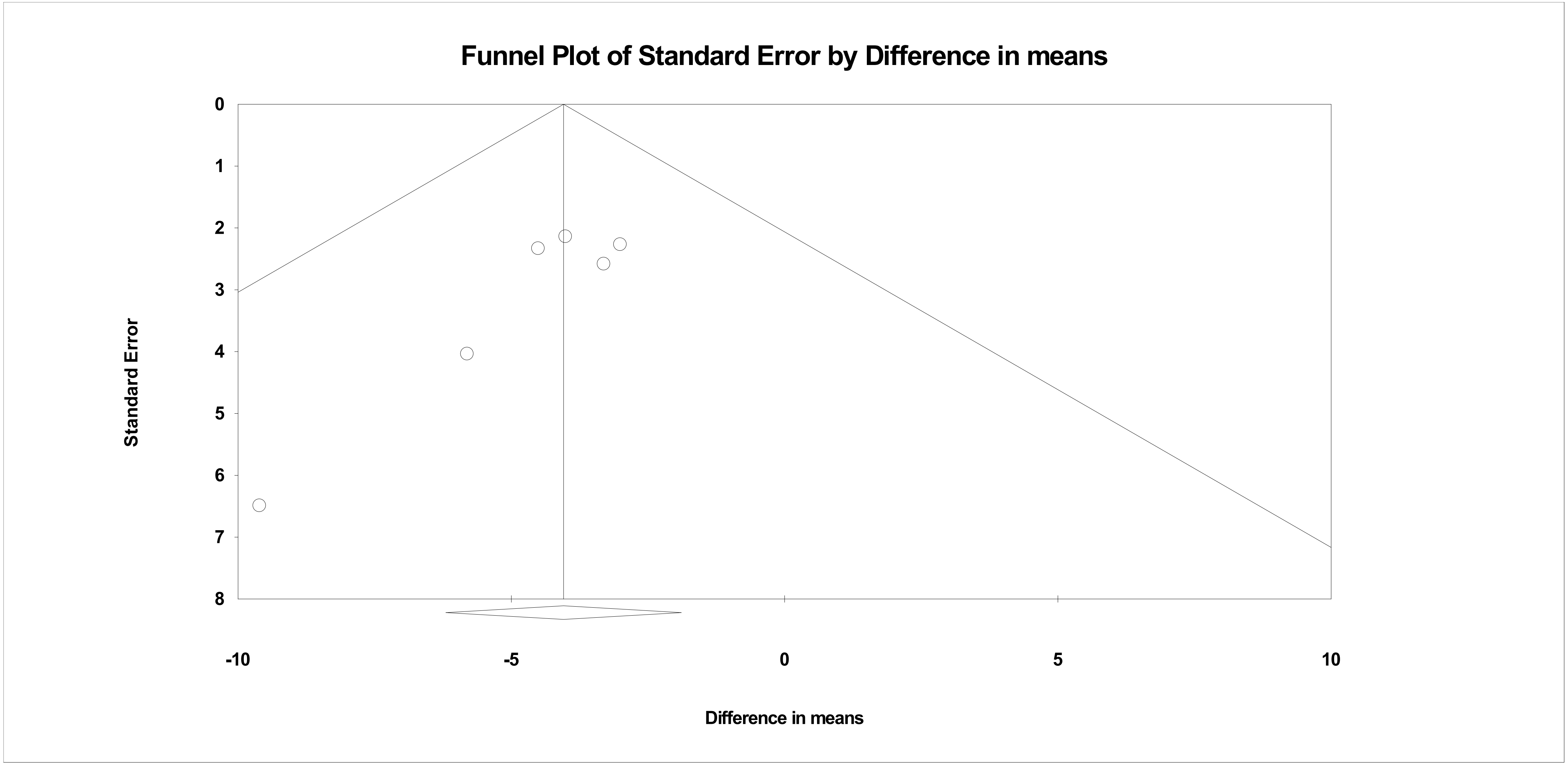

Funnel Plot Asymmetry for E/E'

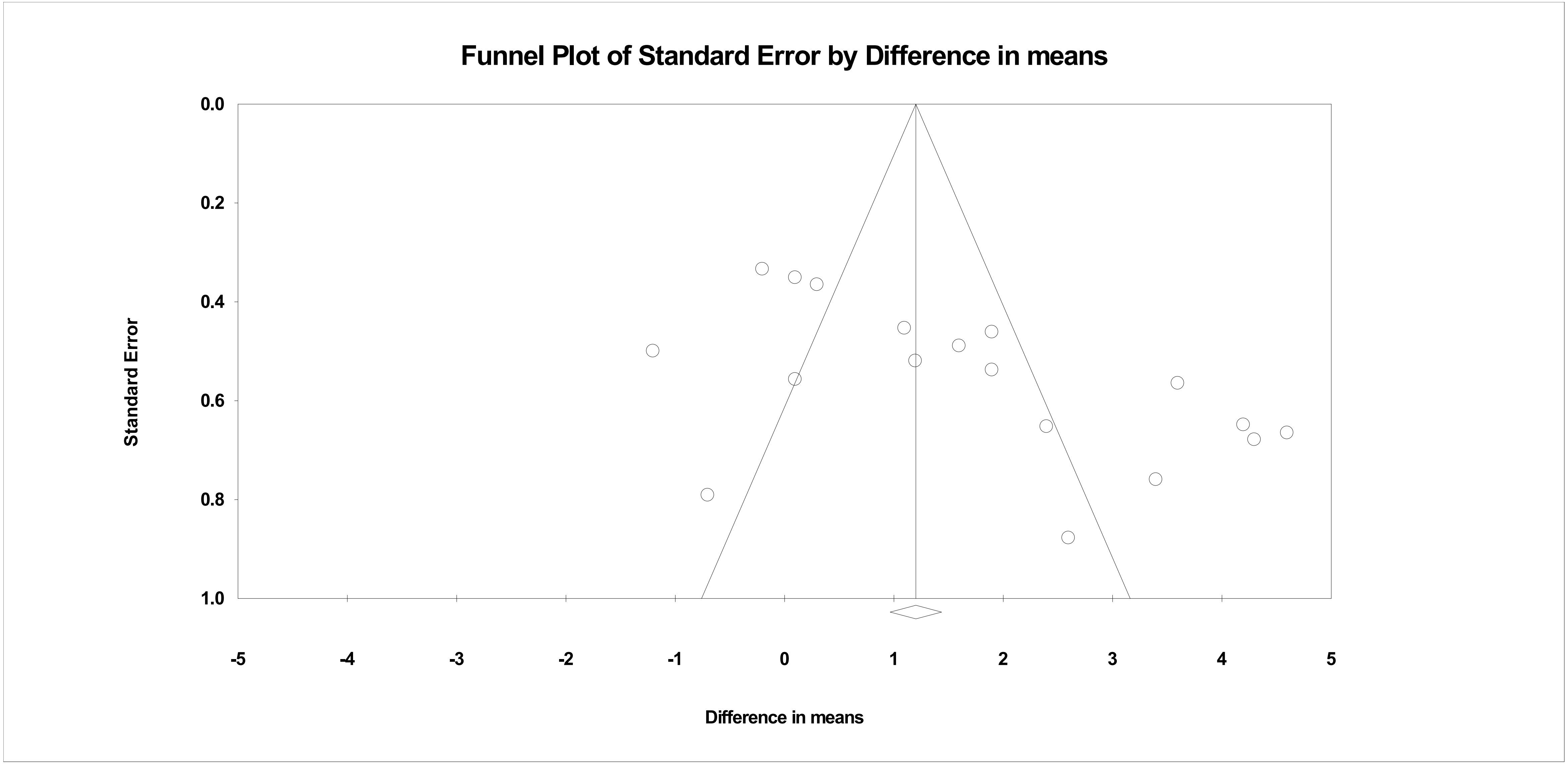

Funnel Plot Asymmetry for RV FWS

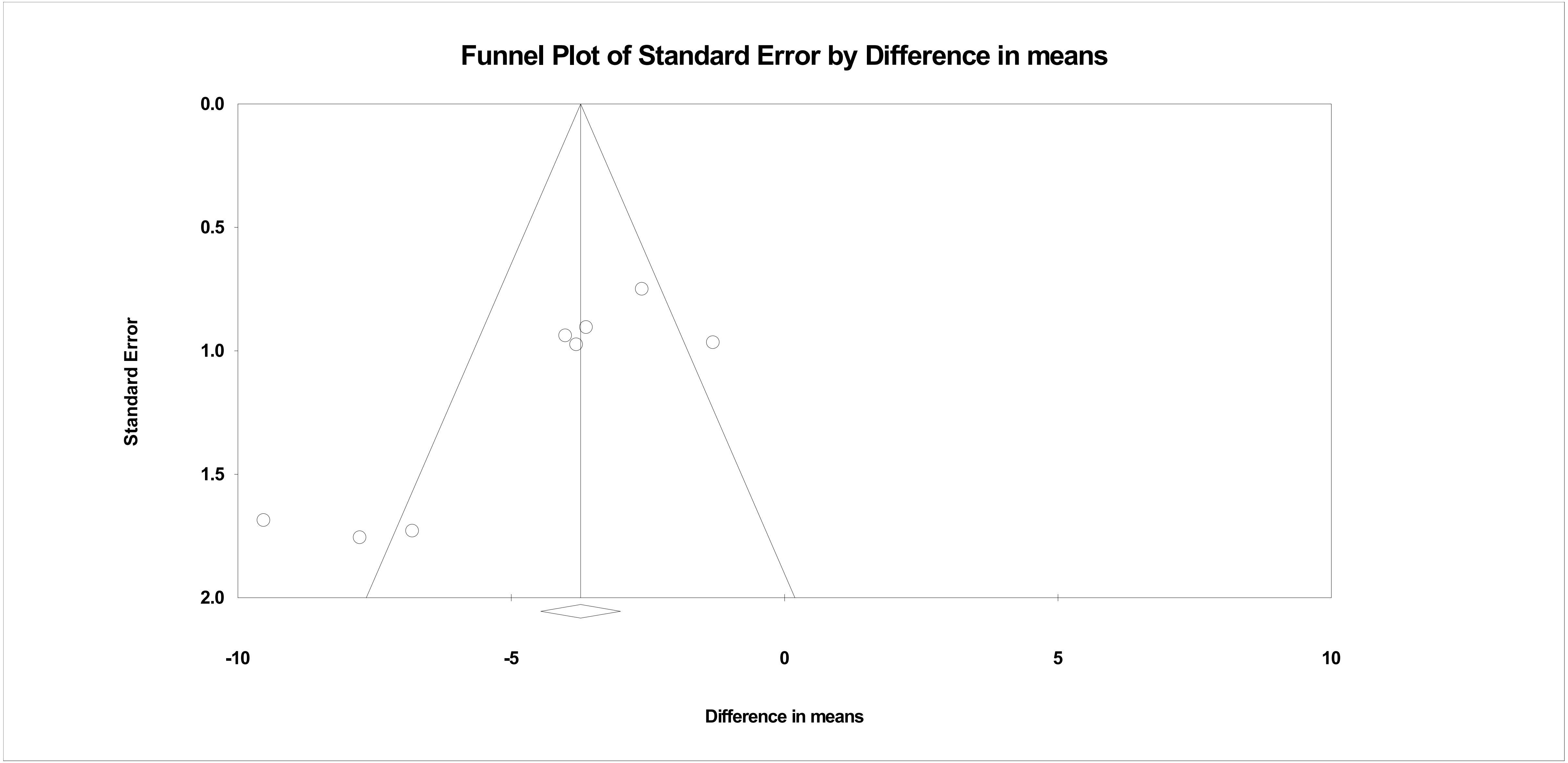

Supplement: Supplementary file 2 — Supplementary Material 2. [file 44156_2025_81_MOESM2_ESM.pdf]
