# Supplementary material for: Cardiac function and mechanics in systemic sclerosis: a systematic review and meta-analysis
Source: Echo Res Pract. 2025 Jul 14;12:18. doi: 10.1186/s44156-025-00081-4 (PMC12257727; doi:10.1186/s44156-025-00081-4)
Supplement: Supplementary file 3 — Supplementary Material 3. [file 44156_2025_81_MOESM3_ESM.pdf]

Title	Year	ASelection 1	ASelection 2	ASelection 3	ASelection 4	ASelection TOTAL	BComparability	BComparability TOTAL	CExposure 1	CExposure 2	CExposure 3	CExposure TOTAL
Left atrial dysfunction detected by speckle tracking in patients with systemic sclerosis	2014	1aStar	2aStar	3c	4b	2Stars	1ab2Stars	2Stars	1aStar	2aStar	3aStar	3Stars
Evaluation of left atrial volume and function in systemic sclerosis patients using speckle tracking and real-time three-dimensional echocardiography	2016	1aStar	2aStar	3c	4b	2Stars	1ab2Stars	2Stars	1aStar	2aStar	3aStar	3Stars
Contractile reserve in systemic sclerosis patients as a major predictor of global cardiac impairment and exercise tolerance	2015	1aStar	2b	3c	4b	1Star	1ab2Stars	2Stars	1aStar	2aStar	3aStar	3Stars
Role of 2D strain in the early identification of left ventricular dysfunction and in the risk stratification of systemic sclerosis patients	2013	1aStar	2aStar	3c	4b	1Star	1ab2Stars	2Stars	1aStar	2aStar	3aStar	3Stars
Myocardial and vascular dysfunction in systemic sclerosis: the potential role of noninvasive assessment in asymptomatic patients	2007	1aStar	2b	3c	4b	1Star	1ab2Stars	2Stars	1aStar	2aStar	3aStar	3Stars
Right atrial morphology and function in patients with systemic sclerosis compared to healthy controls: a two-dimensional strain study	2016	1aStar	2aStar	3c	4b	1Star	1ab2Stars	2Stars	1aStar	2aStar	3aStar	3Stars
Early impairment of myocardial function in systemic sclerosis: non-invasive assessment by Doppler myocardial and strain rate imaging	2005	1aStar	2b	3c	4b	1Star	1ab2Stars	2Stars	1aStar	2aStar	3aStar	3Stars
Associations between left ventricular myocardial involvement and endothelial dysfunction in systemic sclerosis: noninvasive assessment in asymptomatic patients	2007	1aStar	2aStar	3c	4b	2Stars	1ab2Stars	2Stars	1aStar	2aStar	3aStar	3Stars
The evaluation of right ventricle dyssynchrony by speckle tracking echocardiography in systemic sclerosis patients	2021	1aStar	2aStar	3c	4b	2Stars	1ab2Stars	2Stars	1aStar	2aStar	3aStar	3Stars
Right ventricular and atrial functions in systemic sclerosis patients without pulmonary hypertension. Speckle-tracking echocardiographic study	2015	1aStar	2b	3b	4b	1Star	1ab2Stars	2Stars	1aStar	2aStar	3aStar	3Stars
Global longitudinal strain measured by speckle tracking identifies subclinical heart involvement in patients with systemic sclerosis	2018	1aStar	2b	3c	4b	2Stars	1ab2Stars	2Stars	1aStar	2aStar	3aStar	3Stars
The relationship between global longitudinal strain and pulmonary function tests in patients with scleroderma and normal ejection fraction and pulmonary artery pressure: a case-control study	2020	1aStar	2b	3b	4b	2Stars	1ab2Stars	2Stars	1aStar	2aStar	3aStar	3Stars
Myocardial fibrosis detected by magnetic resonance in systemic sclerosis patients - Relationship with biochemical and echocardiography parameters	2017	1aStar	2b	3aStar	4b	1Star	1ab2Stars	2Stars	1aStar	2aStar	3aStar	3Stars
Evaluation of left and right ventricle by two-dimensional speckle tracking echocardiography in systemic sclerosis patients without overt cardiac disease	2020	1aStar	2b	3c	4b	2Stars	1ab2Stars	2Stars	1aStar	2aStar	3aStar	3Stars
Detection of subclinical cardiac involvement in systemic sclerosis by echocardiographic strain imaging	2008	1aStar	2aStar	3c	4b	1Star	1ab2Stars	2Stars	1aStar	2aStar	3aStar	3Stars
Speckle-tracking-derived strain and strain-rate analysis: A technique for the evaluation of early alterations in right ventricle systolic function in patients with systemic sclerosis and normal pulmonary artery pres	2009	1aStar	2b	3c	4b	2Stars	1ab2Stars	2Stars	1aStar	2aStar	3aStar	3Stars
Abnormalities of left ventricular function in asymptomatic patients with systemic sclerosis using Doppler measures of myocardial strain	2008	1aStar	2aStar	3c	4b	2Stars	1ab2Stars	2Stars	1aStar	2aStar	3aStar	3Stars
Essential Hypertension Worsens Left Ventricular Contractility in Systemic Sclerosis	2021	1aStar	2aStar	3c	4b	2Stars	1ab2Stars	2Stars	1aStar	2aStar	3aStar	3Stars
Unique Abnormalities in Right Ventricular Longitudinal Strain in Systemic Sclerosis Patients	2016	1aStar	2b	3b	4b	2Stars	1ab2Stars	2Stars	1aStar	2aStar	3aStar	3Stars
Left and right ventricular functional status in patients suffering from scleroderma with normal pulmonary arterial pressure	2019	1aStar	2b	3c	4b	2Stars	1ab2Stars	2Stars	1aStar	2aStar	3aStar	3Stars
Relation of Right Atrial Mechanics to Functional Capacity in Patients With Systemic Sclerosis	2018	1aStar	2aStar	3aStar	4b	2Stars	1ab2Stars	2Stars	1aStar	2aStar	3aStar	3Stars
Evaluation of right ventricular function performed by 3D-echocardiography in scleroderma patients	2015	1aStar	2b	3c	4b	2Stars	1ab2Stars	2Stars	1aStar	2aStar	3aStar	3Stars
Impairment of Left Atrial Mechanics Is an Early Sign of Myocardial Involvement in Systemic Sclerosis	2018	1aStar	2aStar	3aStar	4b	2Stars	1ab2Stars	2Stars	1aStar	2aStar	3aStar	3Stars
Subclinical biventricular systolic dysfunction in patients with systemic sclerosis	2019	1aStar	2aStar	3b	4b	2Stars	1ab2Stars	2Stars	1aStar	2aStar	3aStar	3Stars
Mechanics and prognostic value of left and right ventricular dysfunction in patients with systemic sclerosis	2018	1aStar	2aStar	3b	4b	2Stars	1ab2Stars	2Stars	1aStar	2aStar	3aStar	3Stars
Early right ventricular systolic dysfunction in patients with systemic sclerosis without pulmonary hypertension: a Doppler Tissue and Speckle Tracking echocardiography study	2010	1aStar	2b	3c	4b	2Stars	1ab2Stars	2Stars	1aStar	2aStar	3aStar	3Stars
Two-dimensional speckle tracking of the left ventricle in patients with systemic sclerosis for an early detection of myocardial involvement	2012	1aStar	2aStar	3c	4b	2Stars	1ab2Stars	2Stars	1aStar	2aStar	3aStar	3Stars
The relationship between left ventricular deformation and heart rate variability in patients with systemic sclerosis: Two- and three-dimensional strain analysis	2017	1aStar	2aStar	3c	4b	2Stars	1ab2Stars	2Stars	1aStar	2aStar	3aStar	3Stars
Systemic sclerosis impacts right heart and cardiac autonomic nervous system	2018	1aStar	2aStar	3c	4b	2Stars	1ab2Stars	2Stars	1aStar	2aStar	3aStar	3Stars
Left atrial phasic function and heart rate variability in patients with systemic sclerosis: A new part of the old puzzle	2017	1aStar	2aStar	3b	4b	2Stars	1ab2Stars	2Stars	1aStar	2aStar	3aStar	3Stars
Left Ventricular Diastolic Dysfunction Predicts Mortality in Patients With Systemic Sclerosis	2018	1aStar	2aStar	3aStar	4b	2Stars	1ab2Stars	2Stars	1aStar	2aStar	3aStar	3Stars
Systolic Dysfunction in Systemic Sclerosis: Prevalence and Prognostic Implications	2019	1aStar	2b	3b	4b	2Stars	1ab2Stars	2Stars	1aStar	2aStar	3aStar	3Stars
Regional myocardial dysfunction assessed by two-dimensional speckle tracking echocardiography in systemic sclerosis patients with fragmented QRS complexes	2014	1aStar	2aStar	3b	4b	2Stars	1ab2Stars	2Stars	1aStar	2aStar	3aStar	3Stars
Mechanics of early ventricular impairment in systemic sclerosis and the effects of peripheral arterial haemodynamics	2019	1aStar	2aStar	3b	4b	2Stars	1ab2Stars	2Stars	1aStar	2aStar	3aStar	3Stars
Impact of pulmonary fibrosis and elevated pulmonary pressures on right ventricular function in patients with systemic sclerosis	2016	1aStar	2aStar	3b	4b	2Stars	1ab2Stars	2Stars	1aStar	2aStar	3aStar	3Stars
Left ventricular dysfunction assessed by speckle-tracking strain analysis in patients with systemic sclerosis: relationship to functional capacity and ventricular arrhythmias	2011	1aStar	2aStar	3b	4b	2Stars	1ab2Stars	2Stars	1aStar	2aStar	3aStar	3Stars
Speckle tracking echocardiography in systemic sclerosis: A useful method for detection of myocardial involvement	2019	1aStar	2b	3b	4b	2Stars	1ab2Stars	2Stars	1aStar	2aStar	3aStar	3Stars
Cardiac mechanics and heart rate variability in patients with systemic sclerosis: the association that we should not miss	2017	1aStar	2aStar	3b	4b	2Stars	1ab2Stars	2Stars	1aStar	2aStar	3aStar	3Stars
Ventricular and atrial function assessment with transthoracic echocardiography in patients with rheumatic inflammatory disease	2022	1aStar	2b	3c	4b	2Stars	1ab2Stars	2Stars	1aStar	2aStar	3aStar	3Stars
Evaluation of the Left Ventricular Function in Patients with Scleroderma with Normal Pulmonary Artery Pressure Using Myocardial Strain Analysis: A Cross-Sectional Study	2022	1aStar	2b	3b	4b	2Stars	1ab2Stars	2Stars	1aStar	2aStar	3aStar	3Stars
Diagnosis of Simultaneous Atrial and Ventricular Mechanical Performance in Patients with Systemic Sclerosis	2022	1aStar	2b	3b	4b	2Stars	1ab2Stars	2Stars	1aStar	2aStar	3aStar	3Stars
