# Supplementary material for: Cardiac function and mechanics in systemic sclerosis: a systematic review and meta-analysis
Source: Echo Res Pract. 2025 Jul 14;12:18. doi: 10.1186/s44156-025-00081-4 (PMC12257727; doi:10.1186/s44156-025-00081-4)
Supplement: Supplementary file 4 — Supplementary Material 4. [file 44156_2025_81_MOESM4_ESM.pptx]

## Slide 1
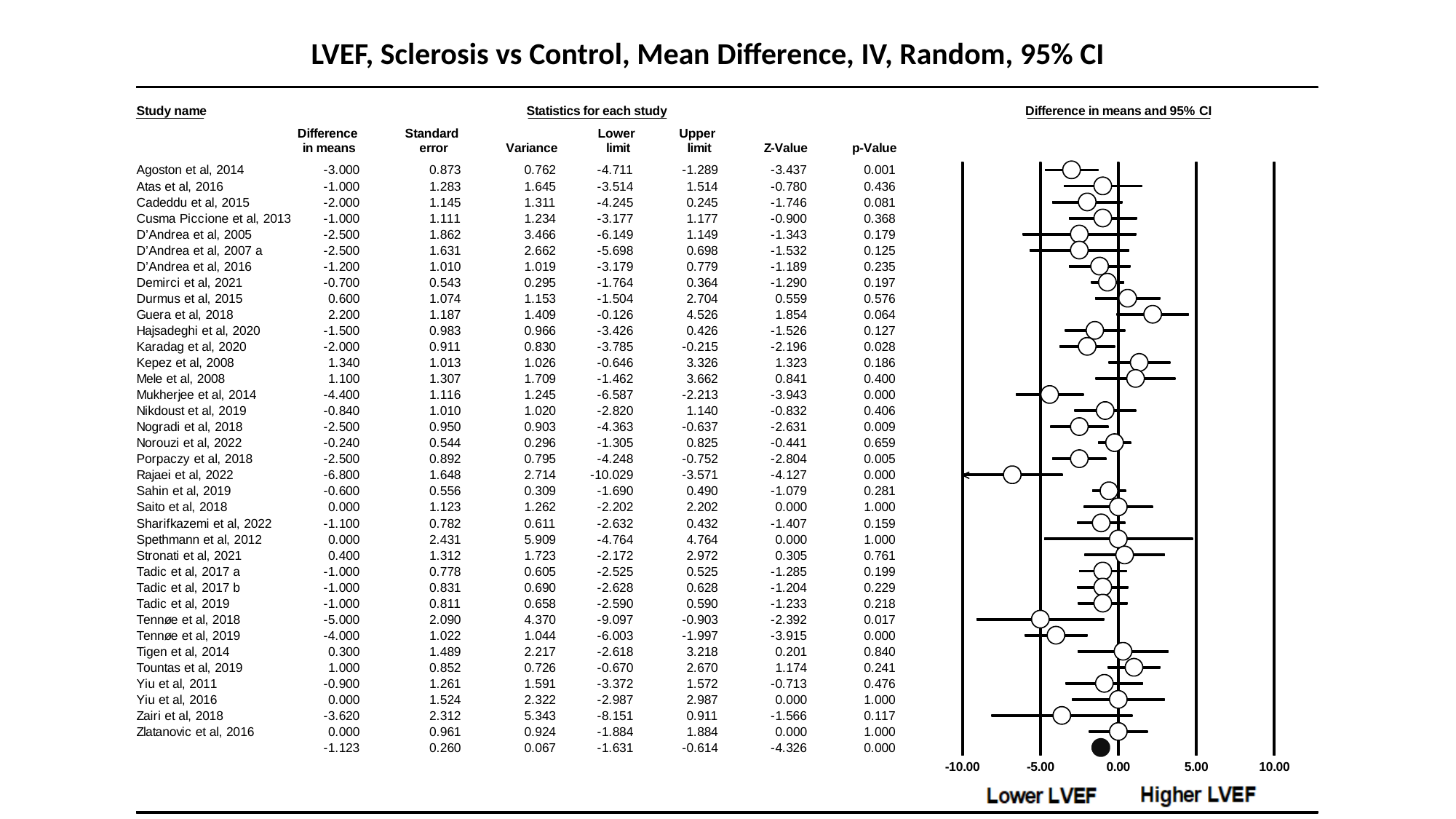

LVEF, Sclerosis vs Control, Mean Difference, IV, Random, 95% CI

## Slide 2
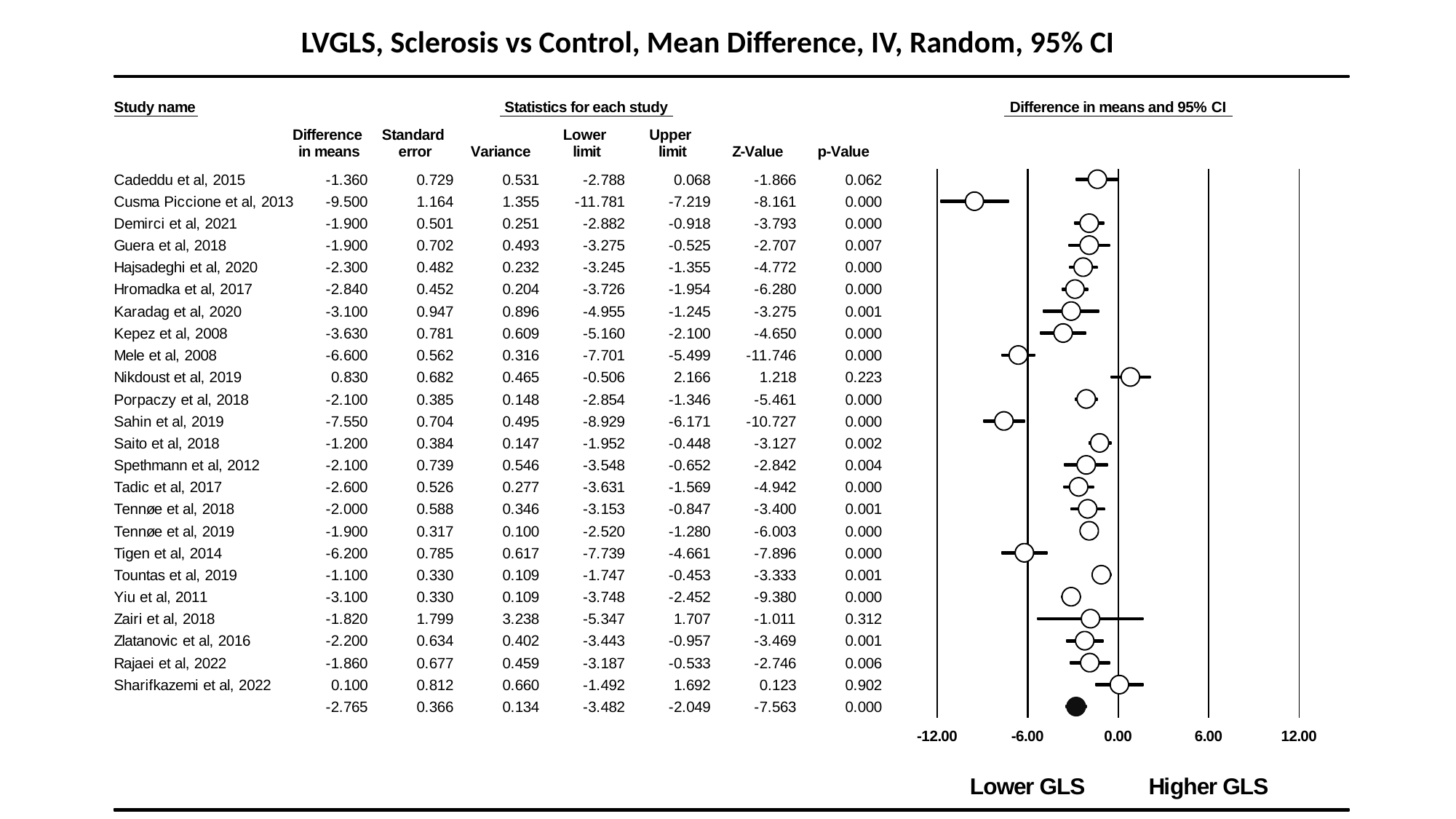

LVGLS, Sclerosis vs Control, Mean Difference, IV, Random, 95% CI

## Slide 3
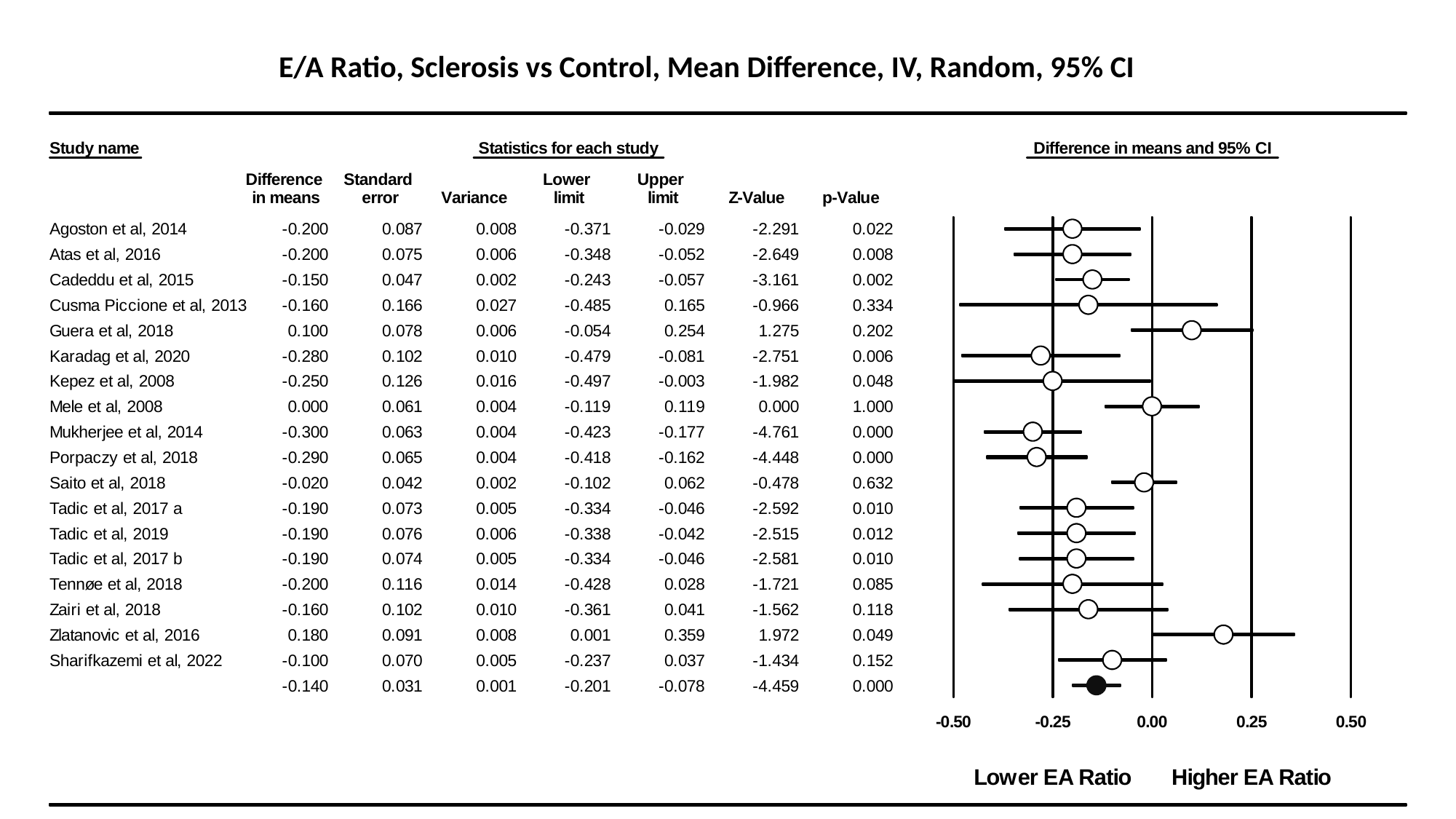

E/A Ratio, Sclerosis vs Control, Mean Difference, IV, Random, 95% CI

## Slide 4
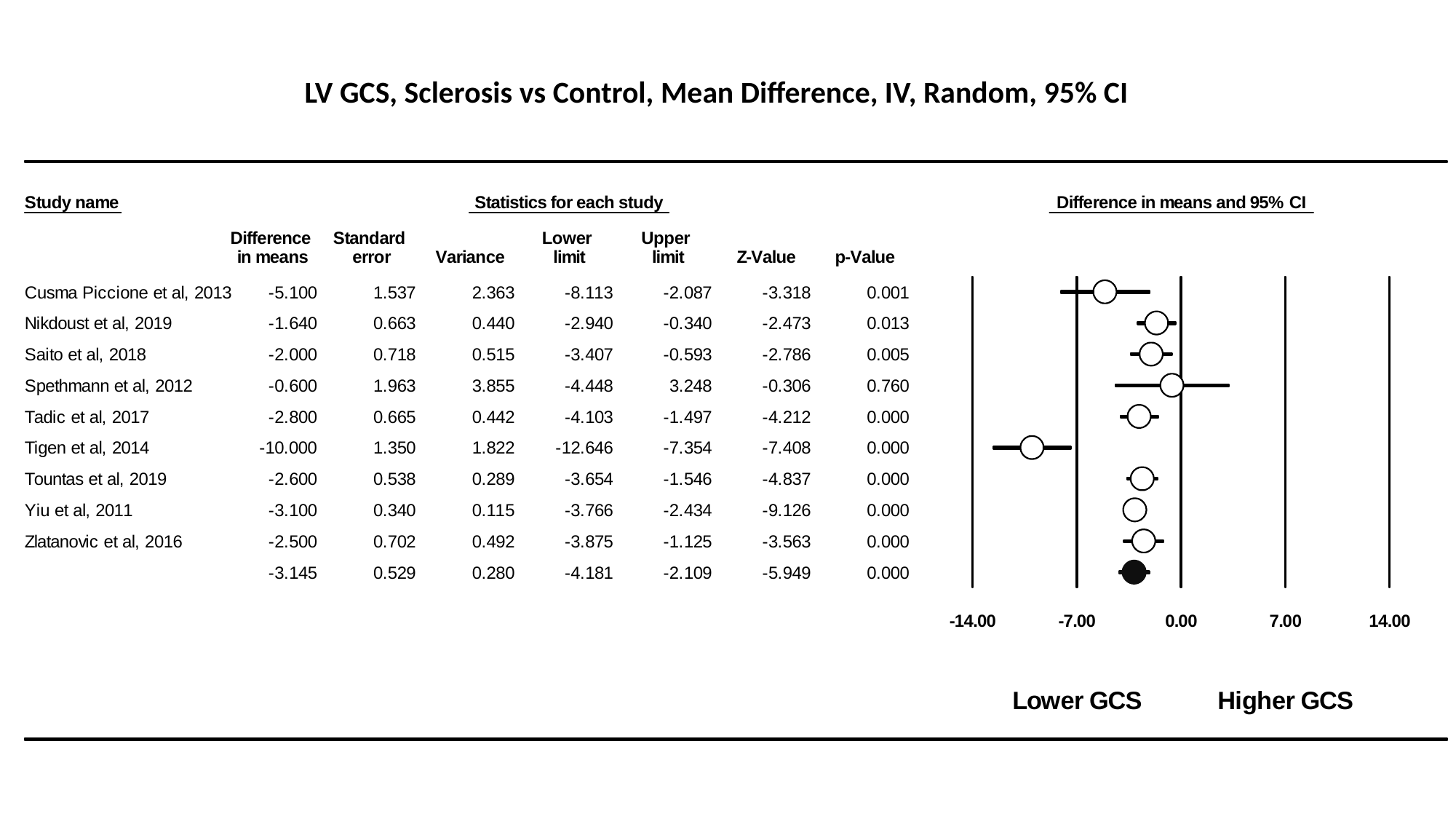

LV GCS, Sclerosis vs Control, Mean Difference, IV, Random, 95% CI

## Slide 5
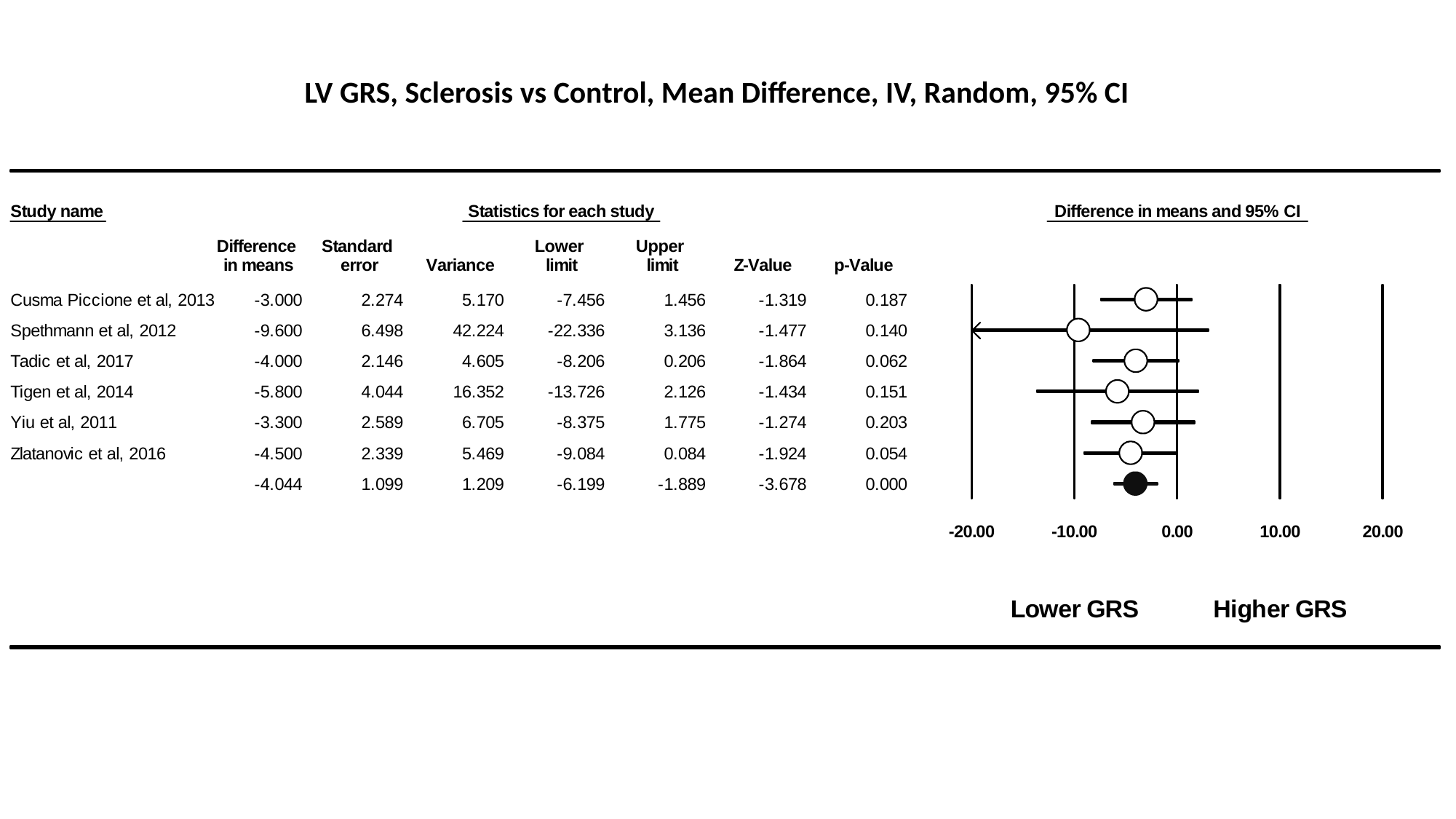

LV GRS, Sclerosis vs Control, Mean Difference, IV, Random, 95% CI

## Slide 6
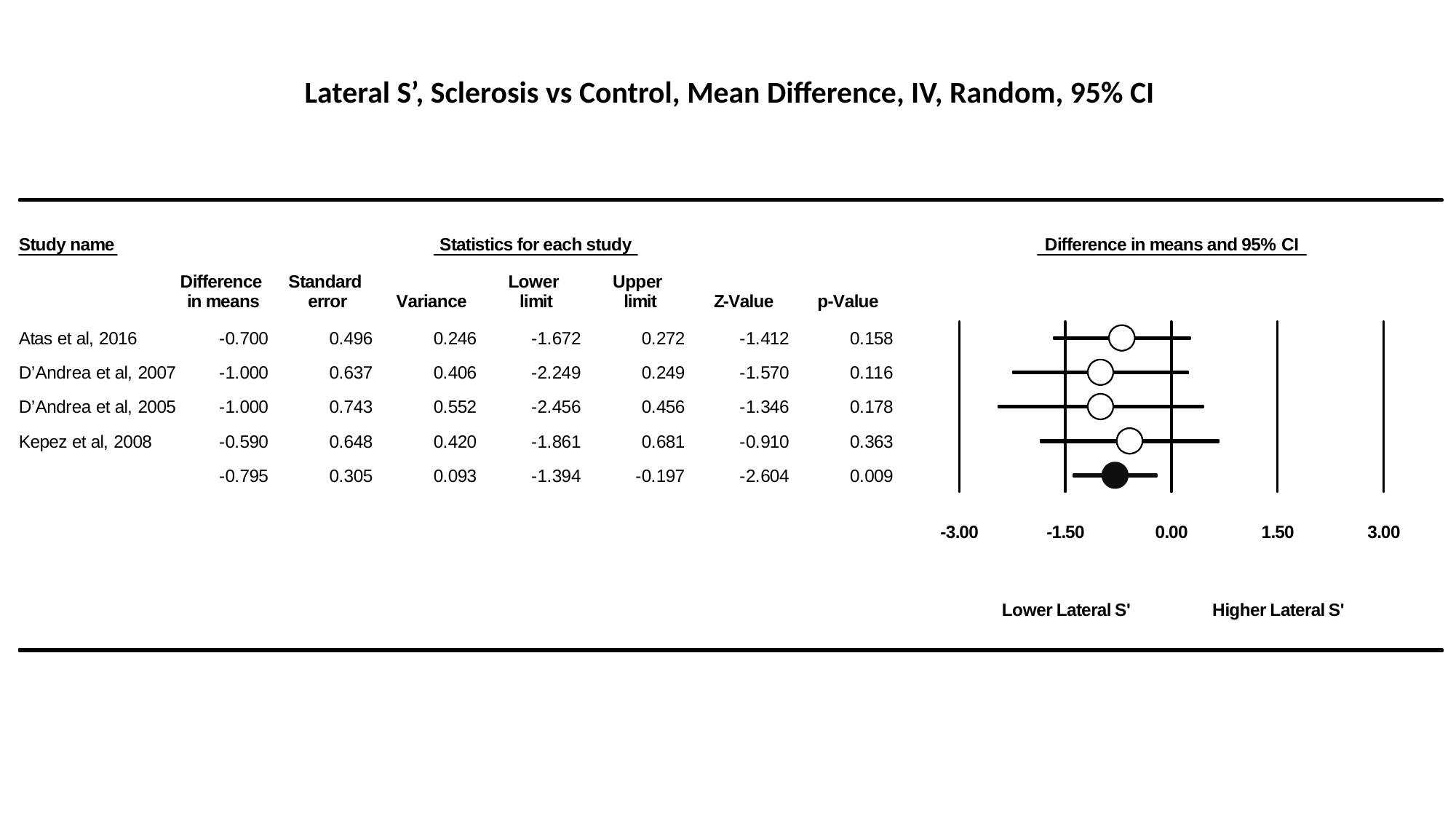

Lateral S’, Sclerosis vs Control, Mean Difference, IV, Random, 95% CI

## Slide 7
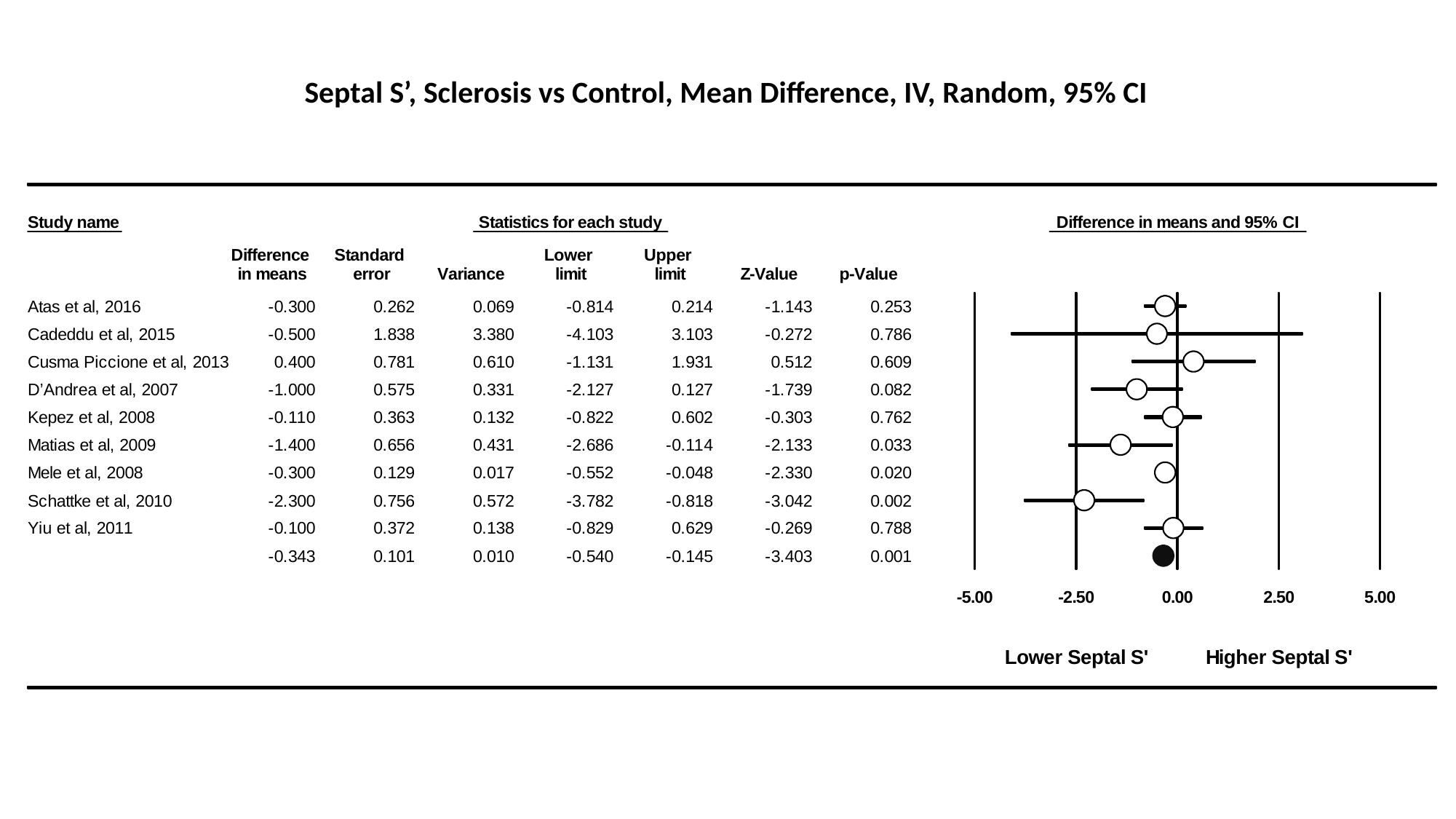

Septal S’, Sclerosis vs Control, Mean Difference, IV, Random, 95% CI

## Slide 8
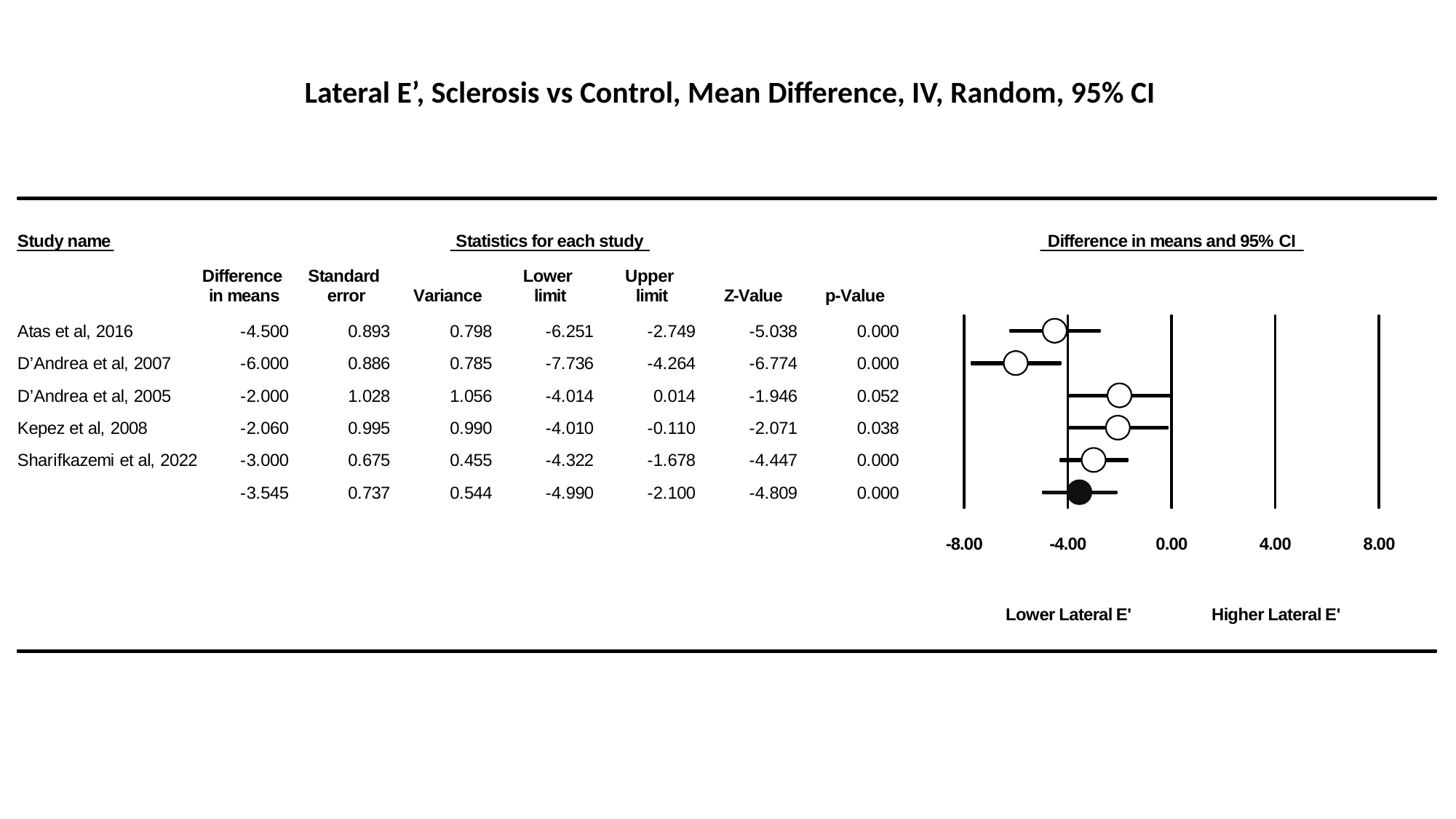

Lateral E’, Sclerosis vs Control, Mean Difference, IV, Random, 95% CI

## Slide 9
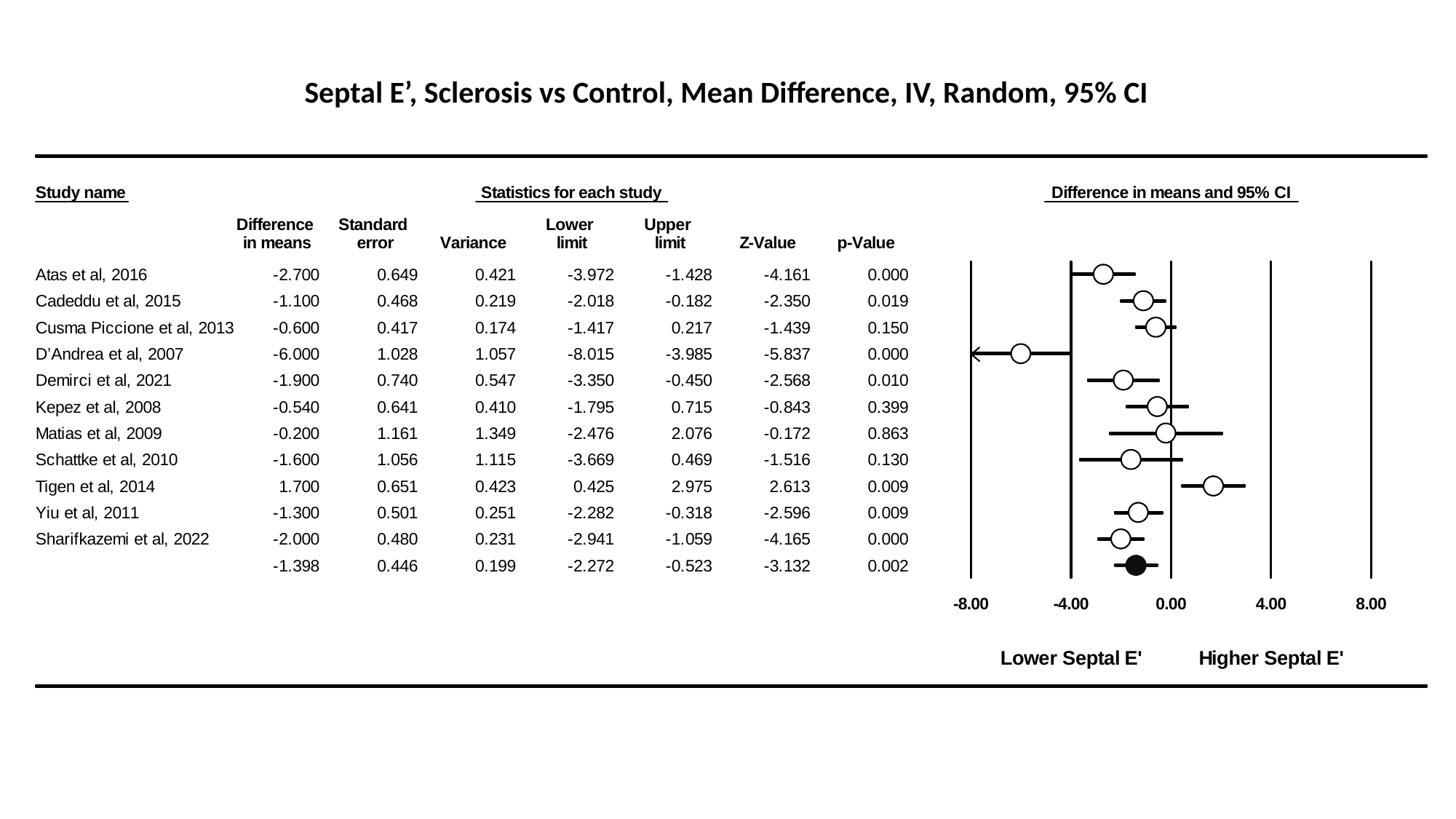

Septal E’, Sclerosis vs Control, Mean Difference, IV, Random, 95% CI

## Slide 10
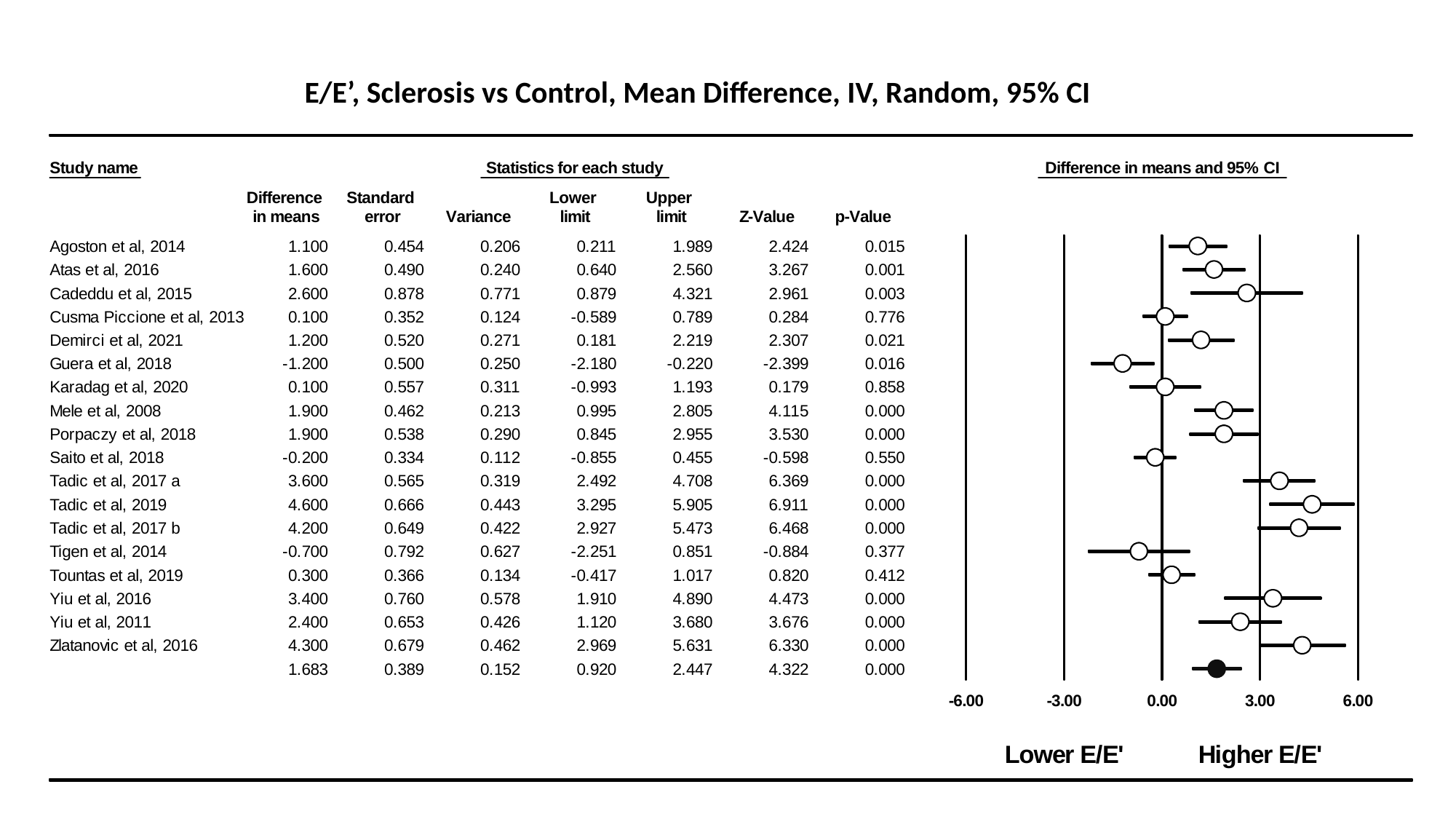

E/E’, Sclerosis vs Control, Mean Difference, IV, Random, 95% CI

## Slide 11
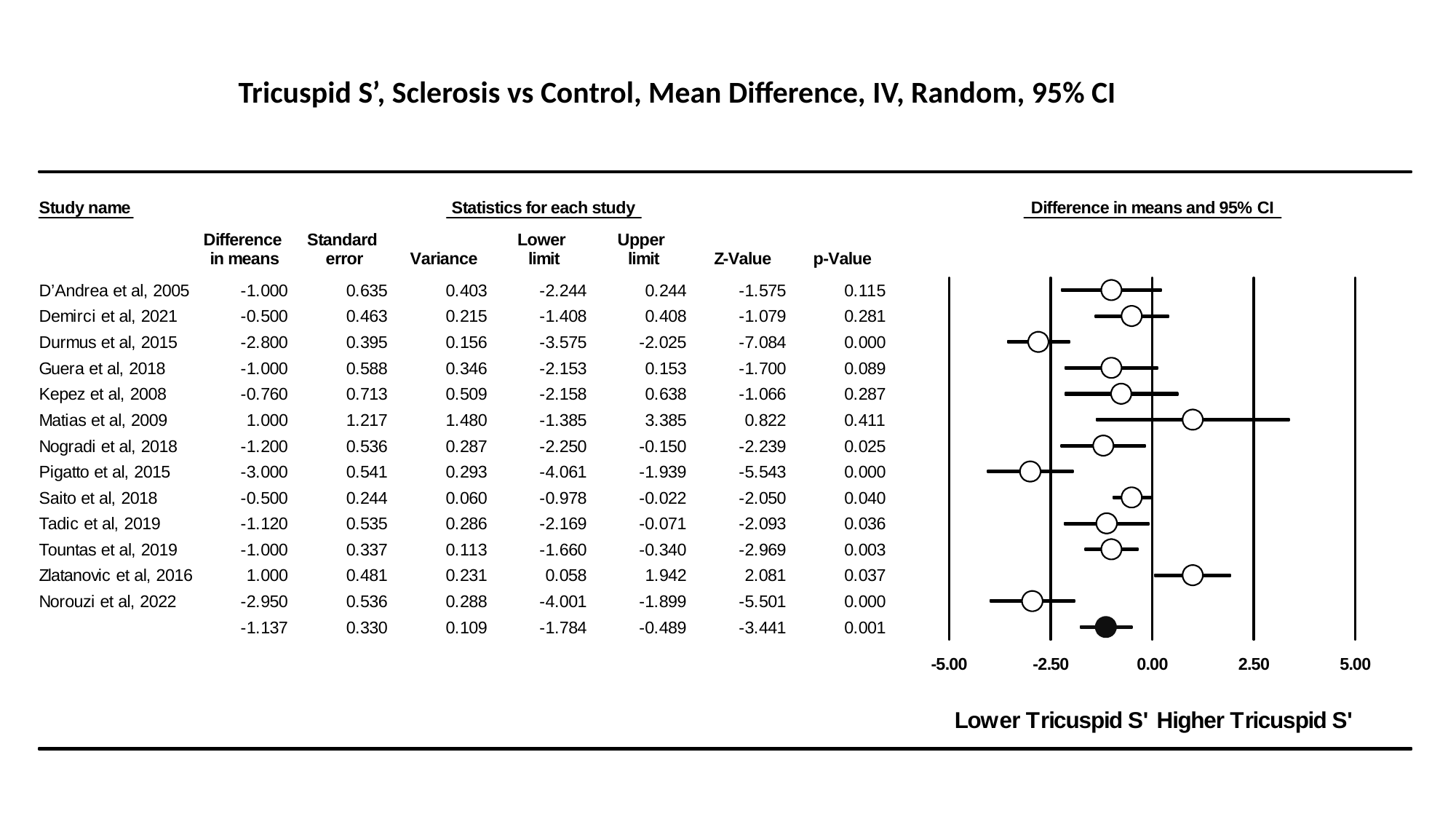

Tricuspid S’, Sclerosis vs Control, Mean Difference, IV, Random, 95% CI

## Slide 12
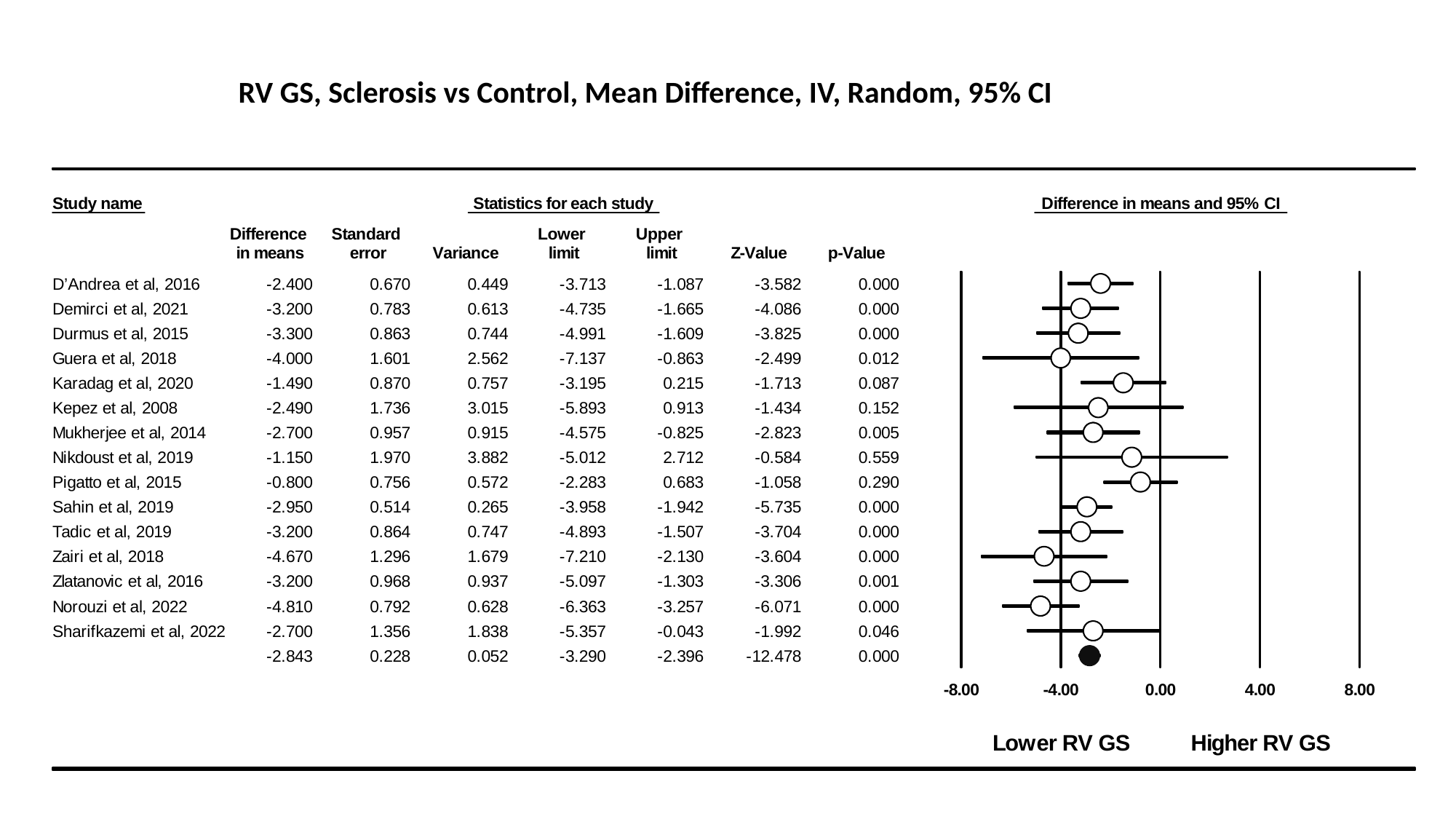

RV GS, Sclerosis vs Control, Mean Difference, IV, Random, 95% CI

## Slide 13
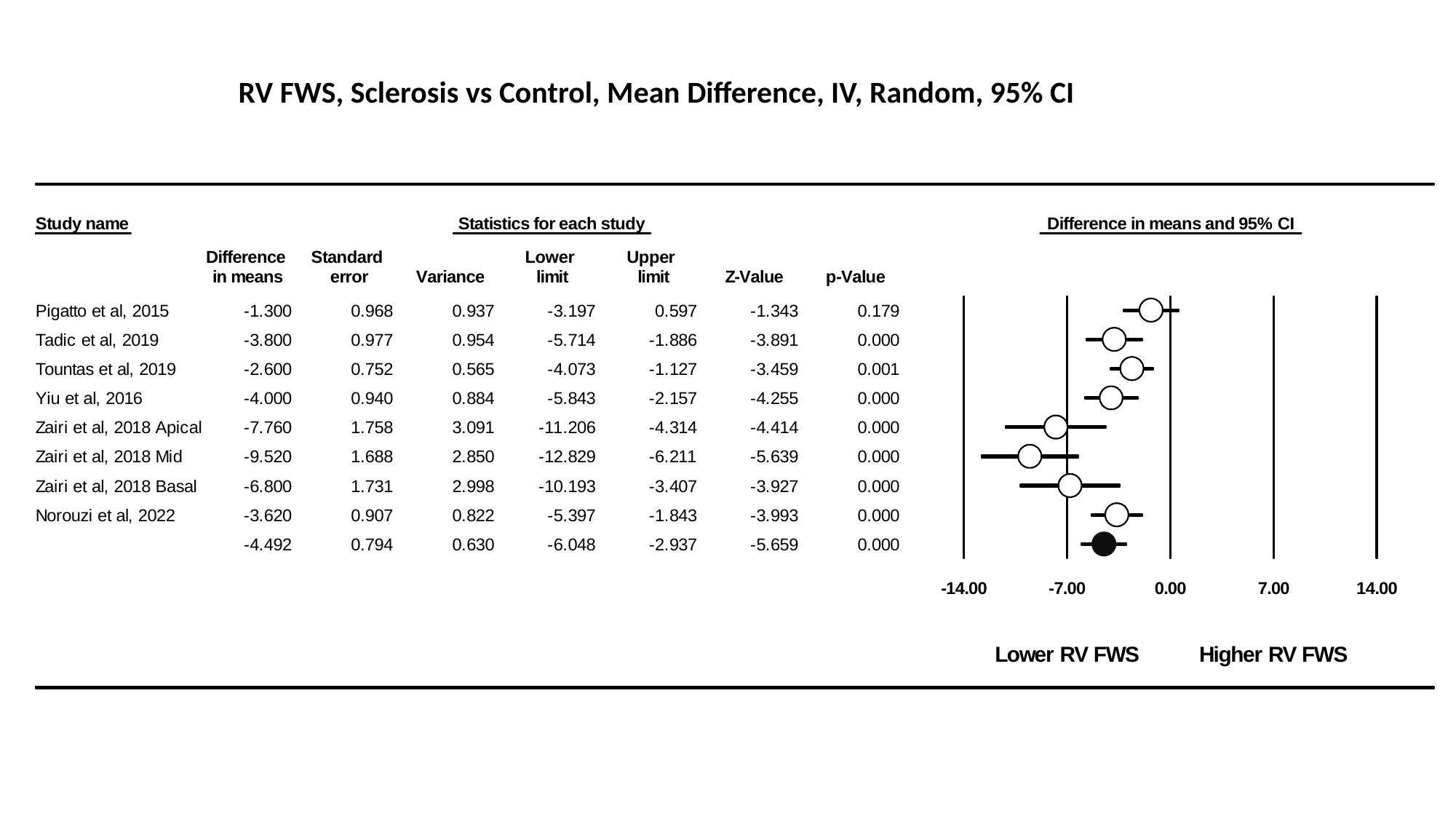

RV FWS, Sclerosis vs Control, Mean Difference, IV, Random, 95% CI

## Slide 14
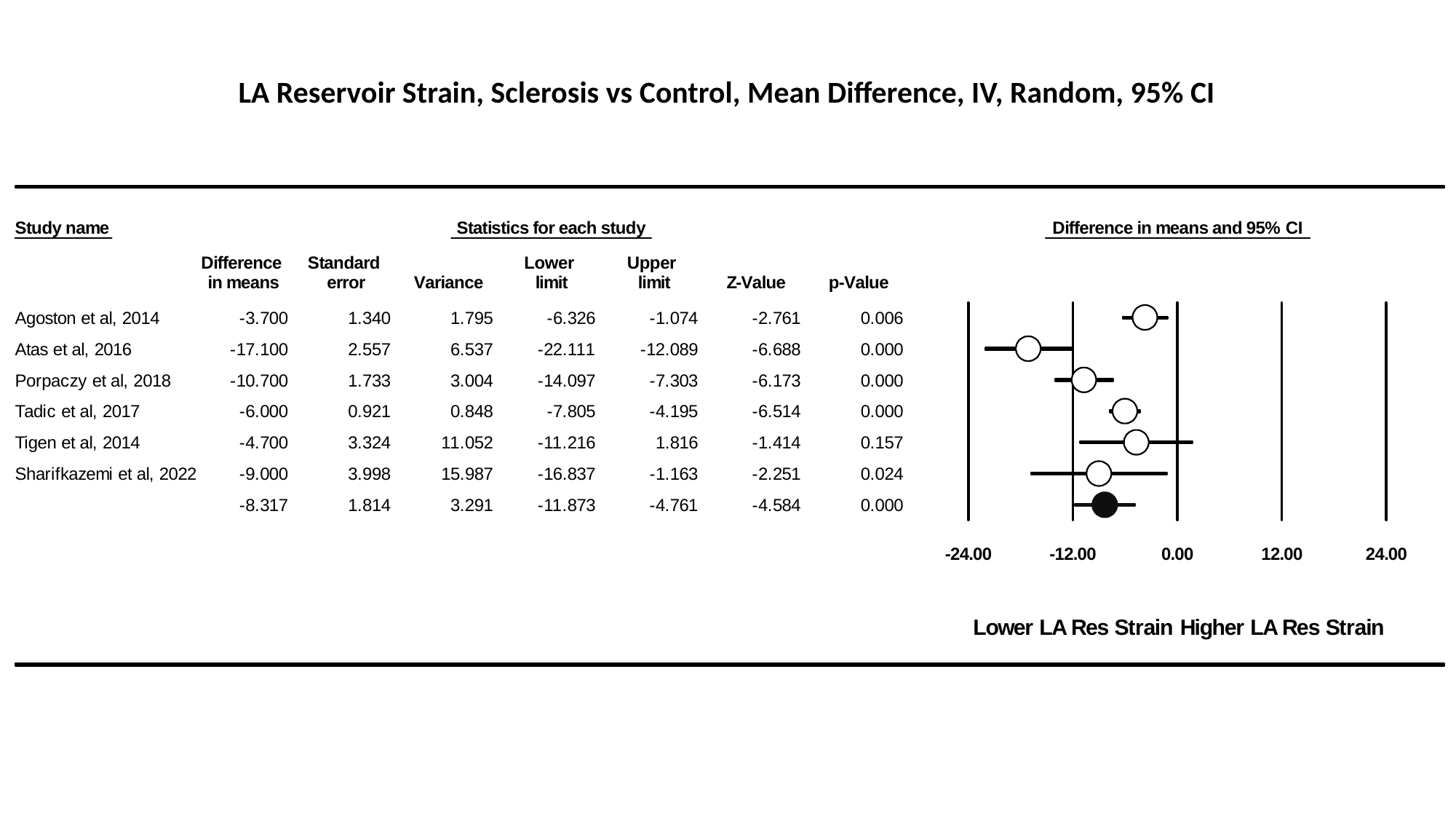

LA Reservoir Strain, Sclerosis vs Control, Mean Difference, IV, Random, 95% CI

## Slide 15
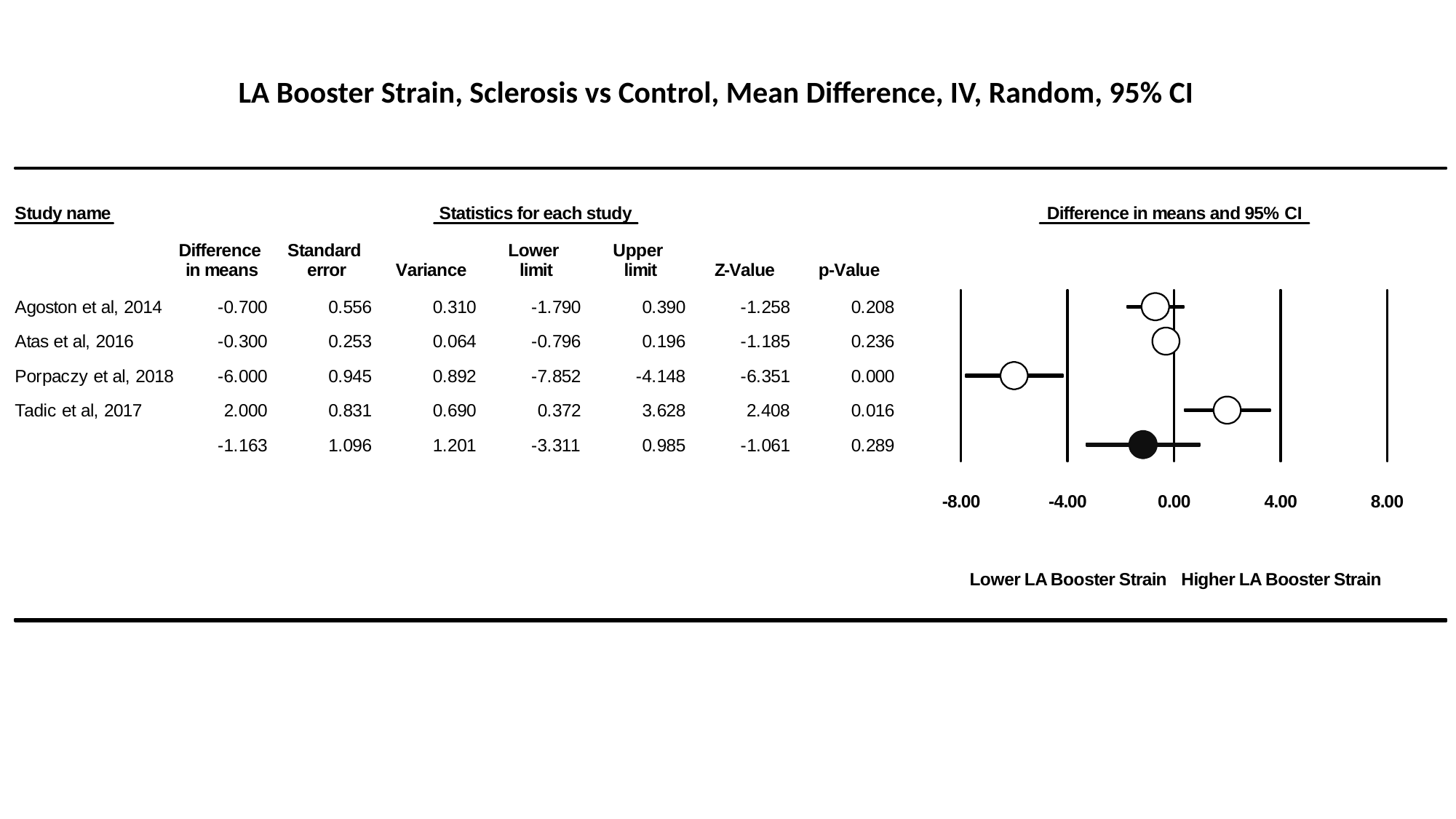

LA Booster Strain, Sclerosis vs Control, Mean Difference, IV, Random, 95% CI

## Slide 16
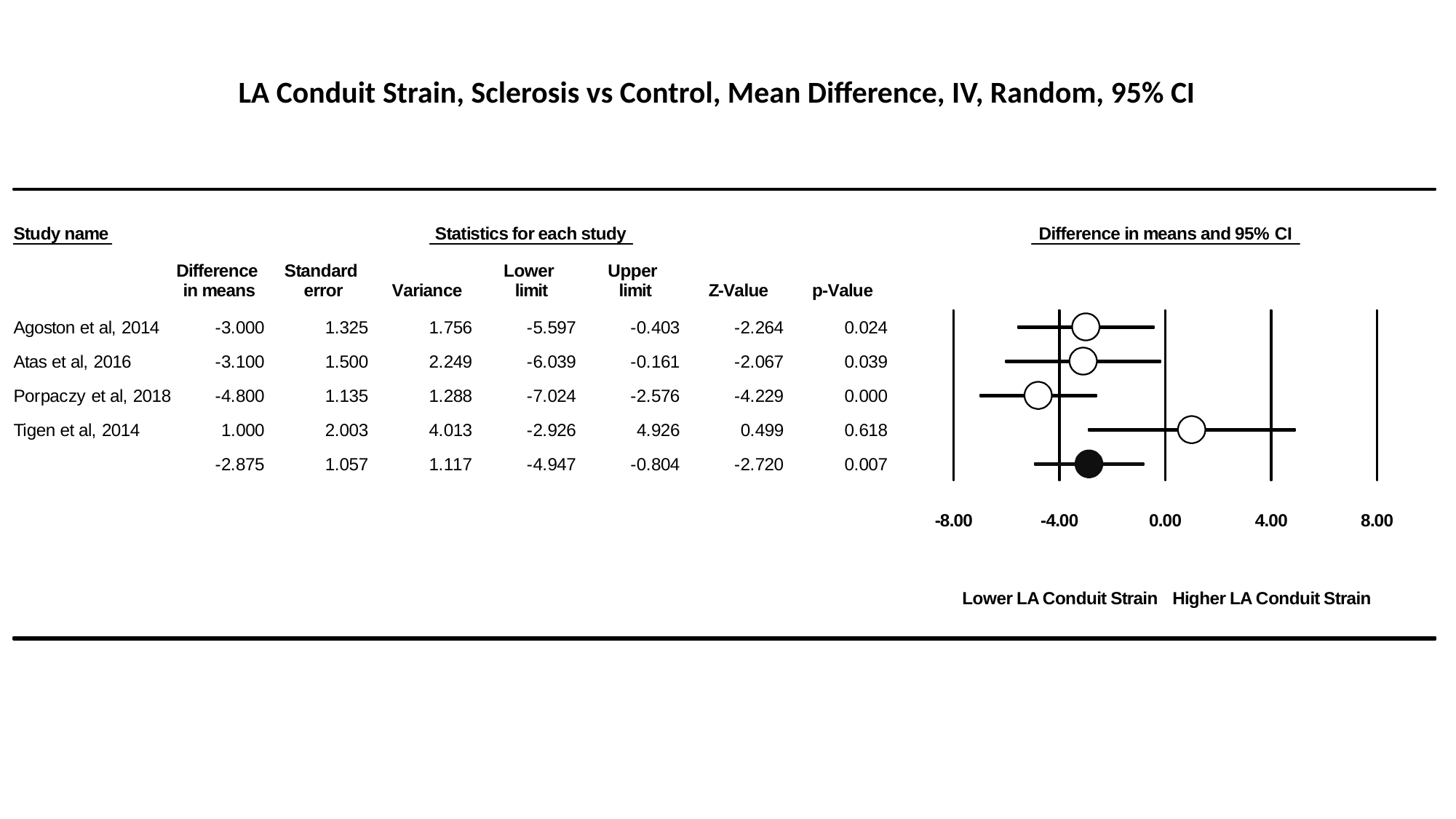

LA Conduit Strain, Sclerosis vs Control, Mean Difference, IV, Random, 95% CI
