# Supplementary material for: Cardiac function and mechanics in systemic sclerosis: a systematic review and meta-analysis
Source: Echo Res Pract. 2025 Jul 14;12:18. doi: 10.1186/s44156-025-00081-4 (PMC12257727; doi:10.1186/s44156-025-00081-4)

LVEF, Sclerosis vs Control, Mean Difference, IV, Random, 95% CI

Study name	Difference in means	Standard error	Variance	Lower limit	Upper limit	Z-Value	p-Value
Agoston et al, 2014	-3.000	0.873	0.762	-4.711	-1.289	-3.437	0.001
Atas et al, 2016	-1.000	1.283	1.645	-3.514	1.514	-0.780	0.436
Cadeddu et al, 2015	-2.000	1.145	1.311	-4.245	0.245	-1.746	0.081
Cusma Piccione et al, 2013	-1.000	1.111	1.234	-3.177	1.177	-0.900	0.368
D’Andrea et al, 2005	-2.500	1.862	3.466	-6.149	1.149	-1.343	0.179
D’Andrea et al, 2007 a	-2.500	1.631	2.662	-5.698	0.698	-1.532	0.125
D’Andrea et al, 2016	-1.200	1.010	1.019	-3.179	0.779	-1.189	0.235
Demirci et al, 2021	-0.700	0.543	0.295	-1.764	0.364	-1.290	0.197
Durmus et al, 2015	0.600	1.074	1.153	-1.504	2.704	0.559	0.576
Guera et al, 2018	2.200	1.187	1.409	-0.126	4.526	1.854	0.064
Hajsadeghi et al, 2020	-1.500	0.983	0.966	-3.426	0.426	-1.526	0.127
Karadag et al, 2020	-2.000	0.911	0.830	-3.785	-0.215	-2.196	0.028
Kepez et al, 2008	1.340	1.013	1.026	-0.646	3.326	1.323	0.186
Mele et al, 2008	1.100	1.307	1.709	-1.462	3.662	0.841	0.400
Mukherjee et al, 2014	-4.400	1.116	1.245	-6.587	-2.213	-3.943	0.000
Nikdoust et al, 2019	-0.840	1.010	1.020	-2.820	1.140	-0.832	0.406
Nogradi et al, 2018	-2.500	0.950	0.903	-4.363	-0.637	-2.631	0.009
Norouzi et al, 2022	-0.240	0.544	0.296	-1.305	0.825	-0.441	0.659
Porpaczy et al, 2018	-2.500	0.892	0.795	-4.248	-0.752	-2.804	0.005
Rajaei et al, 2022	-6.800	1.648	2.714	-10.029	-3.571	-4.127	0.000
Sahin et al, 2019	-0.600	0.556	0.309	-1.690	0.490	-1.079	0.281
Saito et al, 2018	0.000	1.123	1.262	-2.202	2.202	0.000	1.000
Sharifkazemi et al, 2022	-1.100	0.782	0.611	-2.632	0.432	-1.407	0.159
Spethmann et al, 2012	0.000	2.431	5.909	-4.764	4.764	0.000	1.000
Stronati et al, 2021	0.400	1.312	1.723	-2.172	2.972	0.305	0.761
Tadic et al, 2017 a	-1.000	0.778	0.605	-2.525	0.525	-1.285	0.199
Tadic et al, 2017 b	-1.000	0.831	0.690	-2.628	0.628	-1.204	0.229
Tadic et al, 2019	-1.000	0.811	0.658	-2.590	0.590	-1.233	0.218
Tennøe et al, 2018	-5.000	2.090	4.370	-9.097	-0.903	-2.392	0.017
Tennøe et al, 2019	-4.000	1.022	1.044	-6.003	-1.997	-3.915	0.000
Tigen et al, 2014	0.300	1.489	2.217	-2.618	3.218	0.201	0.840
Tountas et al, 2019	1.000	0.852	0.726	-0.670	2.670	1.174	0.241
Yiu et al, 2011	-0.900	1.261	1.591	-3.372	1.572	-0.713	0.476
Yiu et al, 2016	0.000	1.524	2.322	-2.987	2.987	0.000	1.000
Zairi et al, 2018	-3.620	2.312	5.343	-8.151	0.911	-1.566	0.117
Zlatanovic et al, 2016	0.000	0.961	0.924	-1.884	1.884	0.000	1.000
	-1.123	0.260	0.067	-1.631	-0.614	-4.326	0.000

Difference in means and 95% CI

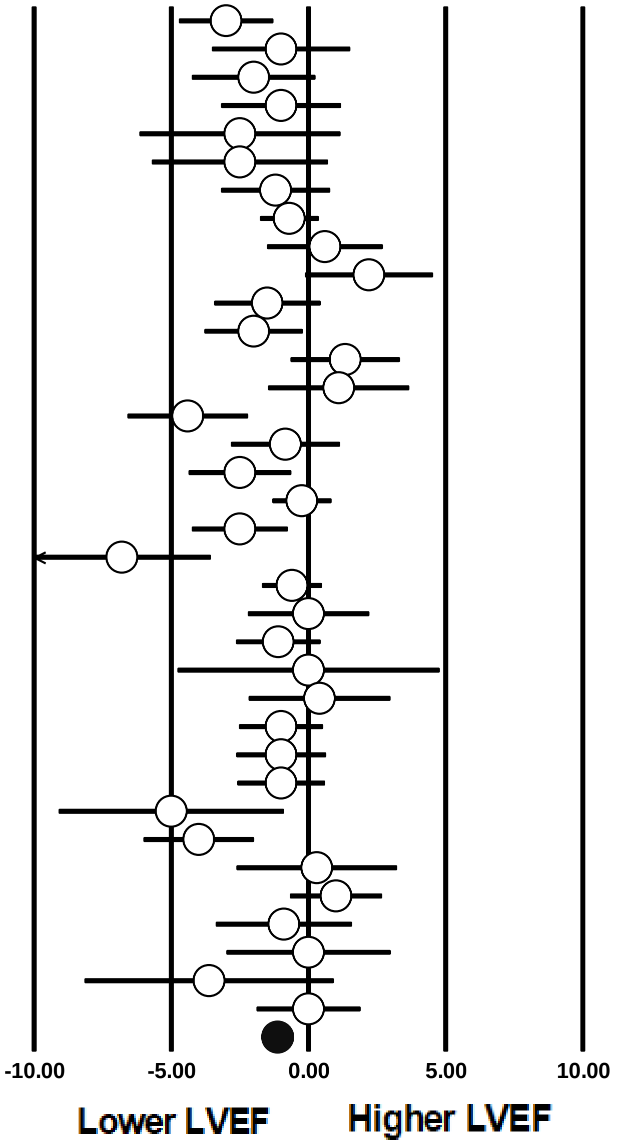

LVGLS, Sclerosis vs Control, Mean Difference, IV, Random, 95% CI

Study name	Difference in means	Standard error	Variance	Lower limit	Upper limit	Z-Value	p-Value
Cadeddu et al, 2015	-1.360	0.729	0.531	-2.788	0.068	-1.866	0.062
Cusma Piccione et al, 2013	-9.500	1.164	1.355	-11.781	-7.219	-8.161	0.000
Demirci et al, 2021	-1.900	0.501	0.251	-2.882	-0.918	-3.793	0.000
Guera et al, 2018	-1.900	0.702	0.493	-3.275	-0.525	-2.707	0.007
Hajsadeghi et al, 2020	-2.300	0.482	0.232	-3.245	-1.355	-4.772	0.000
Hromadka et al, 2017	-2.840	0.452	0.204	-3.726	-1.954	-6.280	0.000
Karadag et al, 2020	-3.100	0.947	0.896	-4.955	-1.245	-3.275	0.001
Kepez et al, 2008	-3.630	0.781	0.609	-5.160	-2.100	-4.650	0.000
Mele et al, 2008	-6.600	0.562	0.316	-7.701	-5.499	-11.746	0.000
Nikdoust et al, 2019	0.830	0.682	0.465	-0.506	2.166	1.218	0.223
Porpaczy et al, 2018	-2.100	0.385	0.148	-2.854	-1.346	-5.461	0.000
Sahin et al, 2019	-7.550	0.704	0.495	-8.929	-6.171	-10.727	0.000
Saito et al, 2018	-1.200	0.384	0.147	-1.952	-0.448	-3.127	0.002
Spethmann et al, 2012	-2.100	0.739	0.546	-3.548	-0.652	-2.842	0.004
Tadic et al, 2017	-2.600	0.526	0.277	-3.631	-1.569	-4.942	0.000
Tennøe et al, 2018	-2.000	0.588	0.346	-3.153	-0.847	-3.400	0.001
Tennøe et al, 2019	-1.900	0.317	0.100	-2.520	-1.280	-6.003	0.000
Tigen et al, 2014	-6.200	0.785	0.617	-7.739	-4.661	-7.896	0.000
Tountas et al, 2019	-1.100	0.330	0.109	-1.747	-0.453	-3.333	0.001
Yiu et al, 2011	-3.100	0.330	0.109	-3.748	-2.452	-9.380	0.000
Zairi et al, 2018	-1.820	1.799	3.238	-5.347	1.707	-1.011	0.312
Zlatanovic et al, 2016	-2.200	0.634	0.402	-3.443	-0.957	-3.469	0.001
Rajaei et al, 2022	-1.860	0.677	0.459	-3.187	-0.533	-2.746	0.006
Sharifkazemi et al, 2022	0.100	0.812	0.660	-1.492	1.692	0.123	0.902
	-2.765	0.366	0.134	-3.482	-2.049	-7.563	0.000

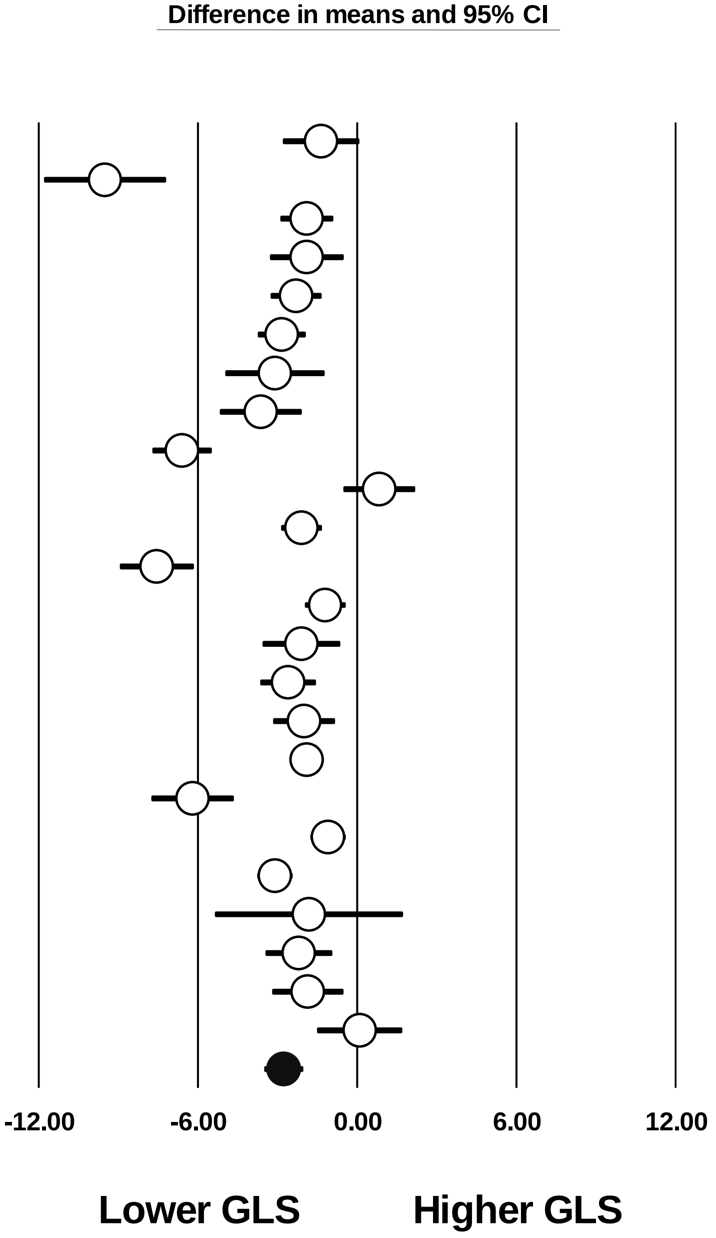

E/A Ratio, Sclerosis vs Control, Mean Difference, IV, Random, 95% CI

Study name	Difference in means	Standard error	Variance	Lower limit	Upper limit	Z-Value	p-Value
Agoston et al, 2014	-0.200	0.087	0.008	-0.371	-0.029	-2.291	0.022
Atas et al, 2016	-0.200	0.075	0.006	-0.348	-0.052	-2.649	0.008
Cadeddu et al, 2015	-0.150	0.047	0.002	-0.243	-0.057	-3.161	0.002
Cusma Piccione et al, 2013	-0.160	0.166	0.027	-0.485	0.165	-0.966	0.334
Guera et al, 2018	0.100	0.078	0.006	-0.054	0.254	1.275	0.202
Karadag et al, 2020	-0.280	0.102	0.010	-0.479	-0.081	-2.751	0.006
Kepez et al, 2008	-0.250	0.126	0.016	-0.497	-0.003	-1.982	0.048
Mele et al, 2008	0.000	0.061	0.004	-0.119	0.119	0.000	1.000
Mukherjee et al, 2014	-0.300	0.063	0.004	-0.423	-0.177	-4.761	0.000
Porpaczy et al, 2018	-0.290	0.065	0.004	-0.418	-0.162	-4.448	0.000
Saito et al, 2018	-0.020	0.042	0.002	-0.102	0.062	-0.478	0.632
Tadic et al, 2017 a	-0.190	0.073	0.005	-0.334	-0.046	-2.592	0.010
Tadic et al, 2019	-0.190	0.076	0.006	-0.338	-0.042	-2.515	0.012
Tadic et al, 2017 b	-0.190	0.074	0.005	-0.334	-0.046	-2.581	0.010
Tennøe et al, 2018	-0.200	0.116	0.014	-0.428	0.028	-1.721	0.085
Zairi et al, 2018	-0.160	0.102	0.010	-0.361	0.041	-1.562	0.118
Zlatanovic et al, 2016	0.180	0.091	0.008	0.001	0.359	1.972	0.049
Sharifkazemi et al, 2022	-0.100	0.070	0.005	-0.237	0.037	-1.434	0.152
	-0.140	0.031	0.001	-0.201	-0.078	-4.459	0.000

Difference in means and 95% CI

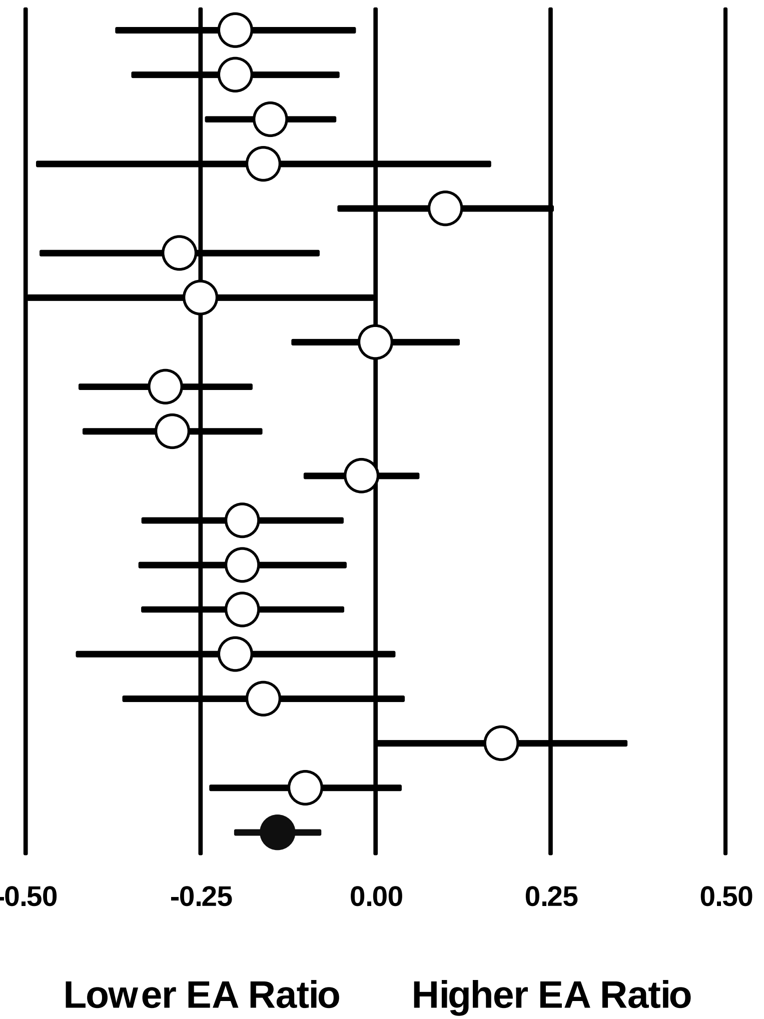

LV GCS, Sclerosis vs Control, Mean Difference, IV, Random, 95% CI

Study name	Difference in means	Standard error	Variance	Lower limit	Upper limit	Z-Value	p-Value
Cusma Piccione et al, 2013	-5.100	1.537	2.363	-8.113	-2.087	-3.318	0.001
Nikdoust et al, 2019	-1.640	0.663	0.440	-2.940	-0.340	-2.473	0.013
Saito et al, 2018	-2.000	0.718	0.515	-3.407	-0.593	-2.786	0.005
Spethmann et al, 2012	-0.600	1.963	3.855	-4.448	3.248	-0.306	0.760
Tadic et al, 2017	-2.800	0.665	0.442	-4.103	-1.497	-4.212	0.000
Tigen et al, 2014	-10.000	1.350	1.822	-12.646	-7.354	-7.408	0.000
Tountas et al, 2019	-2.600	0.538	0.289	-3.654	-1.546	-4.837	0.000
Yiu et al, 2011	-3.100	0.340	0.115	-3.766	-2.434	-9.126	0.000
Zlatanovic et al, 2016	-2.500	0.702	0.492	-3.875	-1.125	-3.563	0.000
	-3.145	0.529	0.280	-4.181	-2.109	-5.949	0.000

Difference in means and 95% CI

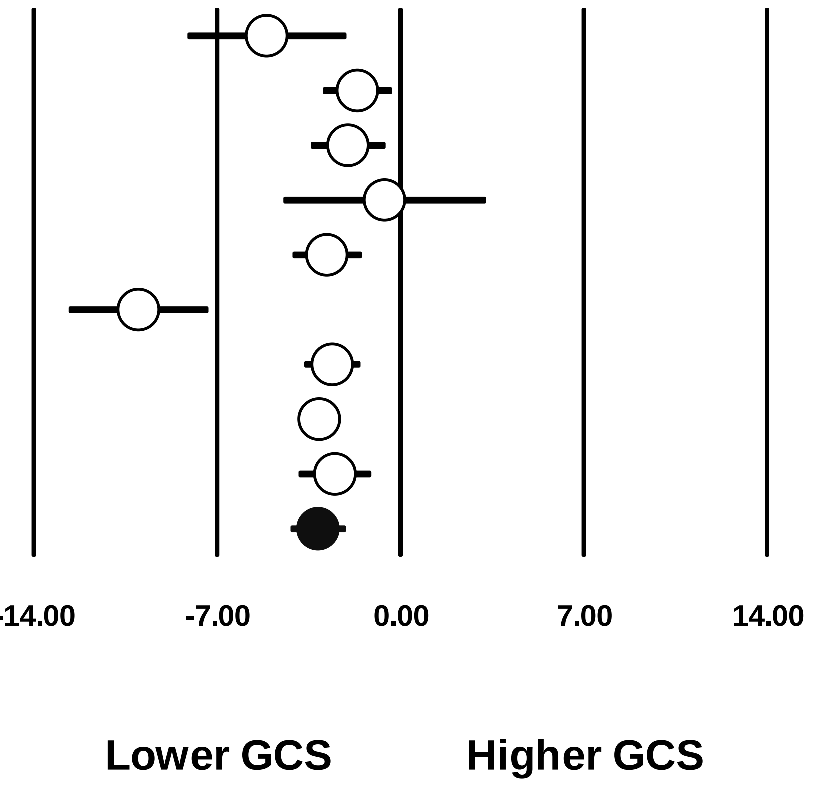

LV GRS, Sclerosis vs Control, Mean Difference, IV, Random, 95% CI

Study name	Difference in means	Standard error	Variance	Lower limit	Upper limit	Z-Value	p-Value
	**Statistics for each study**						
Cusma Piccione et al, 2013	-3.000	2.274	5.170	-7.456	1.456	-1.319	0.187
Spethmann et al, 2012	-9.600	6.498	42.224	-22.336	3.136	-1.477	0.140
Tadic et al, 2017	-4.000	2.146	4.605	-8.206	0.206	-1.864	0.062
Tigen et al, 2014	-5.800	4.044	16.352	-13.726	2.126	-1.434	0.151
Yiu et al, 2011	-3.300	2.589	6.705	-8.375	1.775	-1.274	0.203
Zlatanovic et al, 2016	-4.500	2.339	5.469	-9.084	0.084	-1.924	0.054
	-4.044	1.099	1.209	-6.199	-1.889	-3.678	0.000

Difference in means and 95% CI

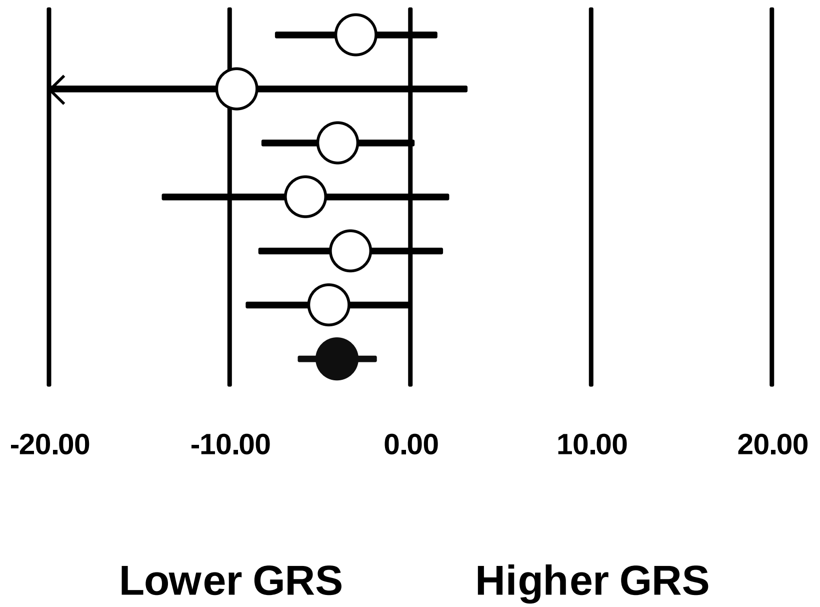

Lateral S’, Sclerosis vs Control, Mean Difference, IV, Random, 95% CI

Study name	Difference in means	Standard error	Variance	Lower limit	Upper limit	Z-Value	p-Value
Atas et al, 2016	-0.700	0.496	0.246	-1.672	0.272	-1.412	0.158
D’Andrea et al, 2007	-1.000	0.637	0.406	-2.249	0.249	-1.570	0.116
D’Andrea et al, 2005	-1.000	0.743	0.552	-2.456	0.456	-1.346	0.178
Kepez et al, 2008	-0.590	0.648	0.420	-1.861	0.681	-0.910	0.363
	-0.795	0.305	0.093	-1.394	-0.197	-2.604	0.009

Statistics for each study

Difference in means and 95% CI

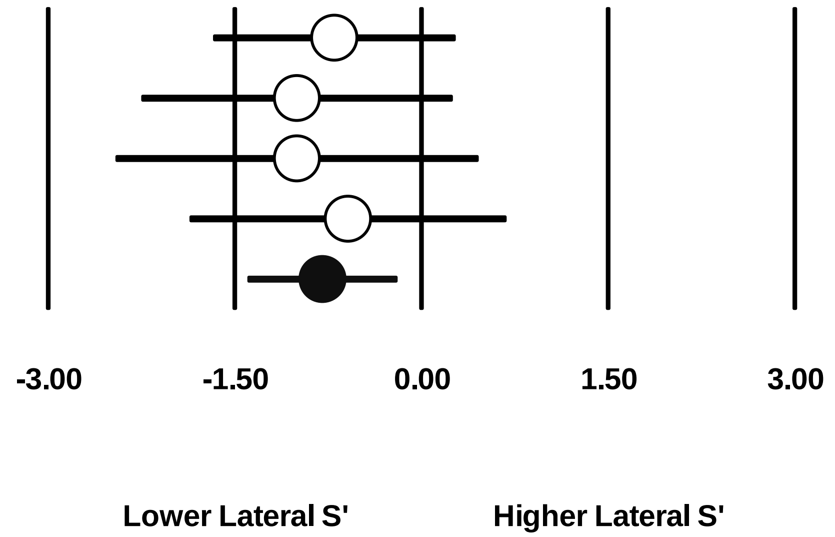

Septal S', Sclerosis vs Control, Mean Difference, IV, Random, 95% CI

Study name	Difference in means	Standard error	Variance	Lower limit	Upper limit	Z-Value	p-Value
	Statistics for each study						
Atas et al, 2016	-0.300	0.262	0.069	-0.814	0.214	-1.143	0.253
Cadeddu et al, 2015	-0.500	1.838	3.380	-4.103	3.103	-0.272	0.786
Cusma Piccione et al, 2013	0.400	0.781	0.610	-1.131	1.931	0.512	0.609
D'Andrea et al, 2007	-1.000	0.575	0.331	-2.127	0.127	-1.739	0.082
Kepez et al, 2008	-0.110	0.363	0.132	-0.822	0.602	-0.303	0.762
Matias et al, 2009	-1.400	0.656	0.431	-2.686	-0.114	-2.133	0.033
Mele et al, 2008	-0.300	0.129	0.017	-0.552	-0.048	-2.330	0.020
Schattke et al, 2010	-2.300	0.756	0.572	-3.782	-0.818	-3.042	0.002
Yiu et al, 2011	-0.100	0.372	0.138	-0.829	0.629	-0.269	0.788
	-0.343	0.101	0.010	-0.540	-0.145	-3.403	0.001

Difference in means and 95% CI

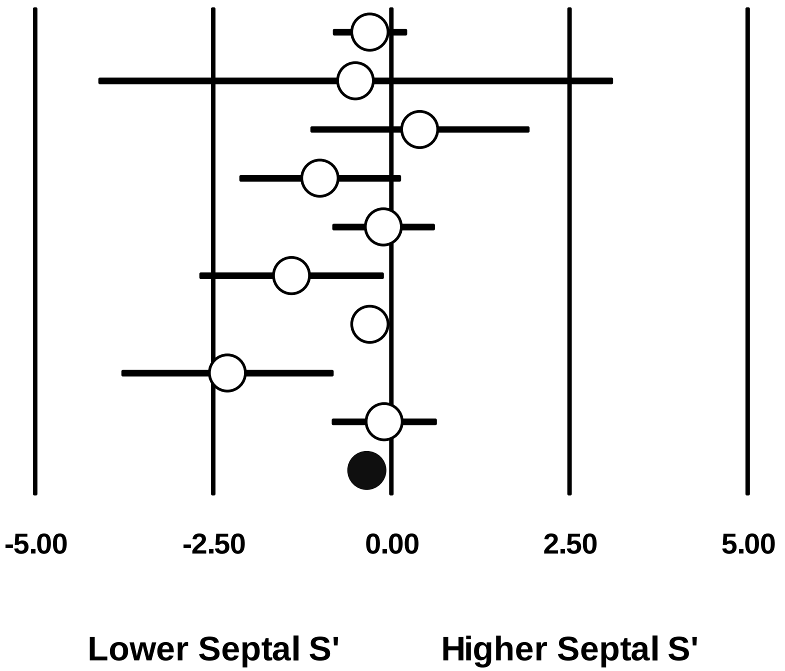

Lateral E’, Sclerosis vs Control, Mean Difference, IV, Random, 95% CI

Study name	Difference in means	Standard error	Variance	Lower limit	Upper limit	Z-Value	p-Value
Atas et al, 2016	-4.500	0.893	0.798	-6.251	-2.749	-5.038	0.000
D’Andrea et al, 2007	-6.000	0.886	0.785	-7.736	-4.264	-6.774	0.000
D’Andrea et al, 2005	-2.000	1.028	1.056	-4.014	0.014	-1.946	0.052
Kepez et al, 2008	-2.060	0.995	0.990	-4.010	-0.110	-2.071	0.038
Sharifkazemi et al, 2022	-3.000	0.675	0.455	-4.322	-1.678	-4.447	0.000
	-3.545	0.737	0.544	-4.990	-2.100	-4.809	0.000

Difference in means and 95% CI

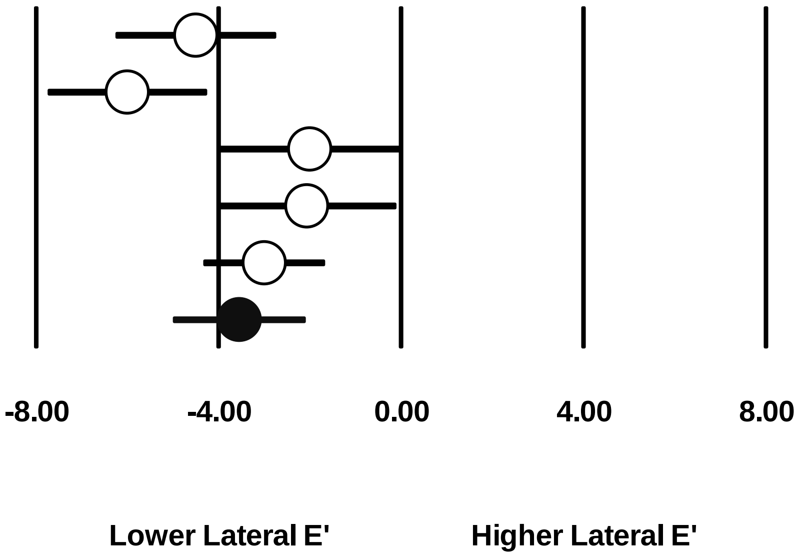

Septal E', Sclerosis vs Control, Mean Difference, IV, Random, 95% CI

Study name	Difference in means	Standard error	Variance	Lower limit	Upper limit	Z-Value	p-Value
Atas et al, 2016	-2.700	0.649	0.421	-3.972	-1.428	-4.161	0.000
Cadeddu et al, 2015	-1.100	0.468	0.219	-2.018	-0.182	-2.350	0.019
Cusma Piccione et al, 2013	-0.600	0.417	0.174	-1.417	0.217	-1.439	0.150
D'Andrea et al, 2007	-6.000	1.028	1.057	-8.015	-3.985	-5.837	0.000
Demirci et al, 2021	-1.900	0.740	0.547	-3.350	-0.450	-2.568	0.010
Kepez et al, 2008	-0.540	0.641	0.410	-1.795	0.715	-0.843	0.399
Matias et al, 2009	-0.200	1.161	1.349	-2.476	2.076	-0.172	0.863
Schattke et al, 2010	-1.600	1.056	1.115	-3.669	0.469	-1.516	0.130
Tigen et al, 2014	1.700	0.651	0.423	0.425	2.975	2.613	0.009
Yiu et al, 2011	-1.300	0.501	0.251	-2.282	-0.318	-2.596	0.009
Sharifkazemi et al, 2022	-2.000	0.480	0.231	-2.941	-1.059	-4.165	0.000
	-1.398	0.446	0.199	-2.272	-0.523	-3.132	0.002

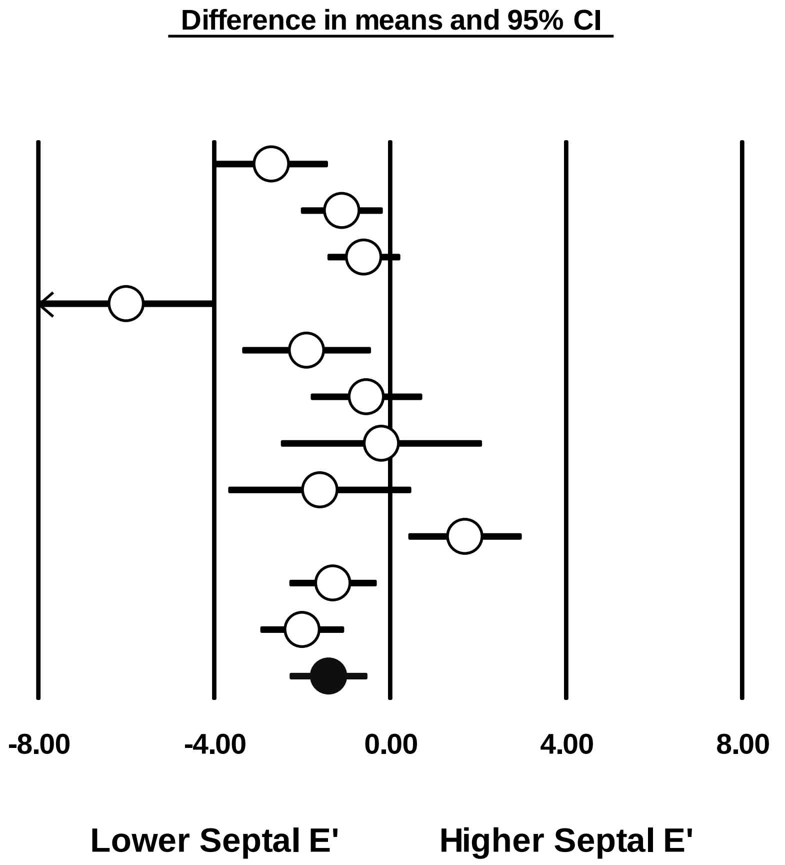

E/E’, Sclerosis vs Control, Mean Difference, IV, Random, 95% CI

Statistics for each study

Study name	Difference in means	Standard error	Variance	Lower limit	Upper limit	Z-Value	p-Value
Agoston et al, 2014	1.100	0.454	0.206	0.211	1.989	2.424	0.015
Atas et al, 2016	1.600	0.490	0.240	0.640	2.560	3.267	0.001
Cadeddu et al, 2015	2.600	0.878	0.771	0.879	4.321	2.961	0.003
Cusma Piccione et al, 2013	0.100	0.352	0.124	-0.589	0.789	0.284	0.776
Demirci et al, 2021	1.200	0.520	0.271	0.181	2.219	2.307	0.021
Guera et al, 2018	-1.200	0.500	0.250	-2.180	-0.220	-2.399	0.016
Karadag et al, 2020	0.100	0.557	0.311	-0.993	1.193	0.179	0.858
Mele et al, 2008	1.900	0.462	0.213	0.995	2.805	4.115	0.000
Porpaczy et al, 2018	1.900	0.538	0.290	0.845	2.955	3.530	0.000
Saito et al, 2018	-0.200	0.334	0.112	-0.855	0.455	-0.598	0.550
Tadic et al, 2017 a	3.600	0.565	0.319	2.492	4.708	6.369	0.000
Tadic et al, 2019	4.600	0.666	0.443	3.295	5.905	6.911	0.000
Tadic et al, 2017 b	4.200	0.649	0.422	2.927	5.473	6.468	0.000
Tigen et al, 2014	-0.700	0.792	0.627	-2.251	0.851	-0.884	0.377
Tountas et al, 2019	0.300	0.366	0.134	-0.417	1.017	0.820	0.412
Yiu et al, 2016	3.400	0.760	0.578	1.910	4.890	4.473	0.000
Yiu et al, 2011	2.400	0.653	0.426	1.120	3.680	3.676	0.000
Zlatanovic et al, 2016	4.300	0.679	0.462	2.969	5.631	6.330	0.000
	1.683	0.389	0.152	0.920	2.447	4.322	0.000

Difference in means and 95% CI

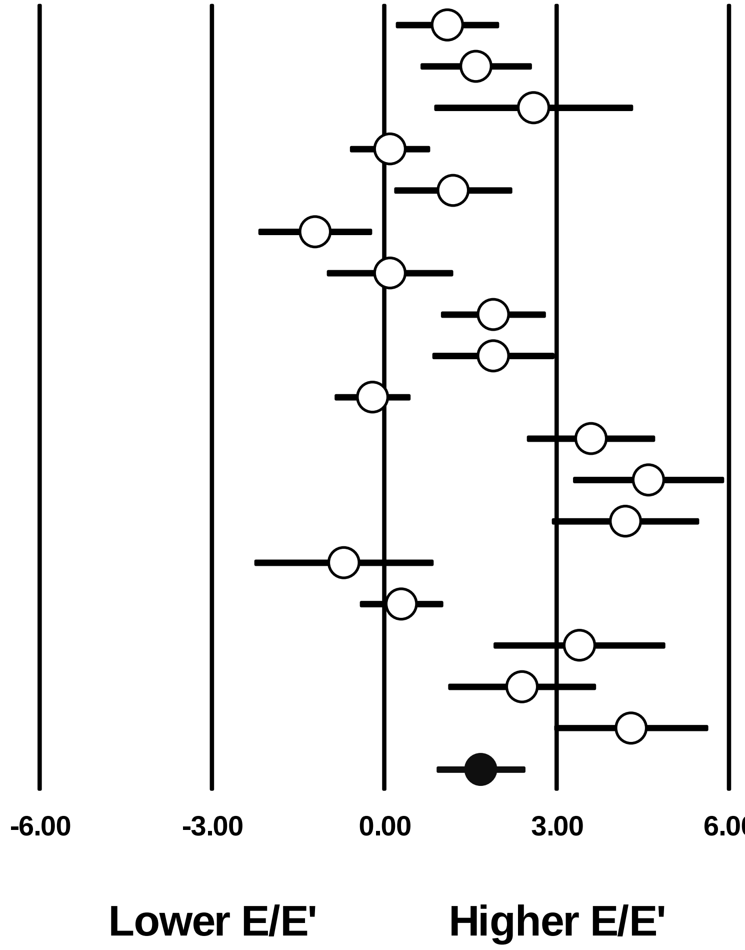

Tricuspid S', Sclerosis vs Control, Mean Difference, IV, Random, 95% CI

Study name	Difference in means	Standard error	Variance	Lower limit	Upper limit	Z-Value	p-Value
D'Andrea et al, 2005	-1.000	0.635	0.403	-2.244	0.244	-1.575	0.115
Demirci et al, 2021	-0.500	0.463	0.215	-1.408	0.408	-1.079	0.281
Durmus et al, 2015	-2.800	0.395	0.156	-3.575	-2.025	-7.084	0.000
Guera et al, 2018	-1.000	0.588	0.346	-2.153	0.153	-1.700	0.089
Kepez et al, 2008	-0.760	0.713	0.509	-2.158	0.638	-1.066	0.287
Matias et al, 2009	1.000	1.217	1.480	-1.385	3.385	0.822	0.411
Nogradi et al, 2018	-1.200	0.536	0.287	-2.250	-0.150	-2.239	0.025
Pigatto et al, 2015	-3.000	0.541	0.293	-4.061	-1.939	-5.543	0.000
Saito et al, 2018	-0.500	0.244	0.060	-0.978	-0.022	-2.050	0.040
Tadic et al, 2019	-1.120	0.535	0.286	-2.169	-0.071	-2.093	0.036
Tountas et al, 2019	-1.000	0.337	0.113	-1.660	-0.340	-2.969	0.003
Zlatanovic et al, 2016	1.000	0.481	0.231	0.058	1.942	2.081	0.037
Norouzi et al, 2022	-2.950	0.536	0.288	-4.001	-1.899	-5.501	0.000
	-1.137	0.330	0.109	-1.784	-0.489	-3.441	0.001

Difference in means and 95% CI

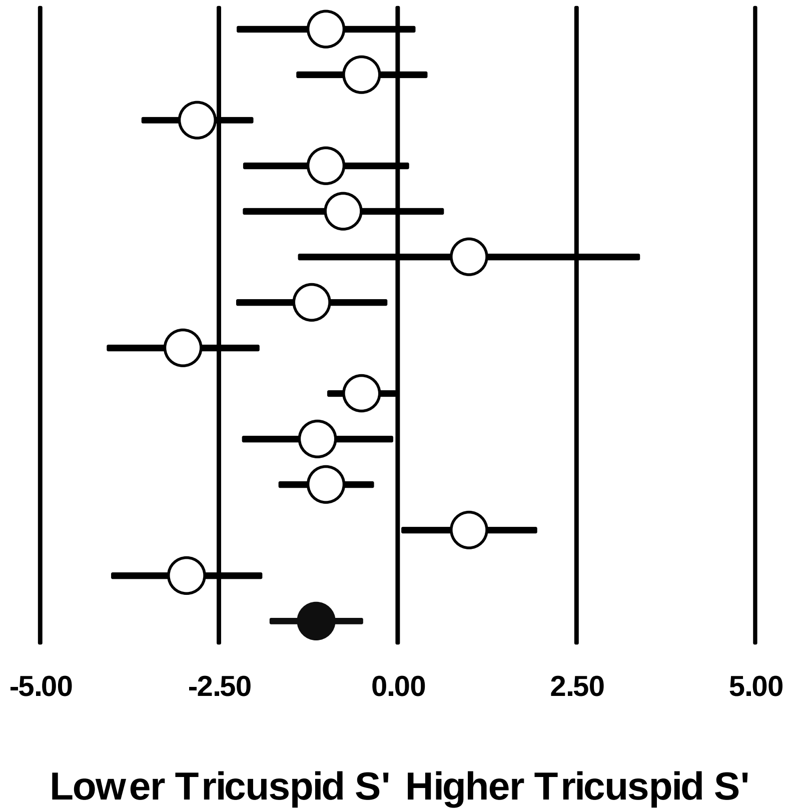

RV GS, Sclerosis vs Control, Mean Difference, IV, Random, 95% CI

Study name	Difference in means	Standard error	Variance	Lower limit	Upper limit	Z-Value	p-Value
	**Statistics for each study**						
D’Andrea et al, 2016	-2.400	0.670	0.449	-3.713	-1.087	-3.582	0.000
Demirci et al, 2021	-3.200	0.783	0.613	-4.735	-1.665	-4.086	0.000
Durmus et al, 2015	-3.300	0.863	0.744	-4.991	-1.609	-3.825	0.000
Guera et al, 2018	-4.000	1.601	2.562	-7.137	-0.863	-2.499	0.012
Karadag et al, 2020	-1.490	0.870	0.757	-3.195	0.215	-1.713	0.087
Kepez et al, 2008	-2.490	1.736	3.015	-5.893	0.913	-1.434	0.152
Mukherjee et al, 2014	-2.700	0.957	0.915	-4.575	-0.825	-2.823	0.005
Nikdoust et al, 2019	-1.150	1.970	3.882	-5.012	2.712	-0.584	0.559
Pigatto et al, 2015	-0.800	0.756	0.572	-2.283	0.683	-1.058	0.290
Sahin et al, 2019	-2.950	0.514	0.265	-3.958	-1.942	-5.735	0.000
Tadic et al, 2019	-3.200	0.864	0.747	-4.893	-1.507	-3.704	0.000
Zairi et al, 2018	-4.670	1.296	1.679	-7.210	-2.130	-3.604	0.000
Zlatanovic et al, 2016	-3.200	0.968	0.937	-5.097	-1.303	-3.306	0.001
Norouzi et al, 2022	-4.810	0.792	0.628	-6.363	-3.257	-6.071	0.000
Sharifkazemi et al, 2022	-2.700	1.356	1.838	-5.357	-0.043	-1.992	0.046
	-2.843	0.228	0.052	-3.290	-2.396	-12.478	0.000

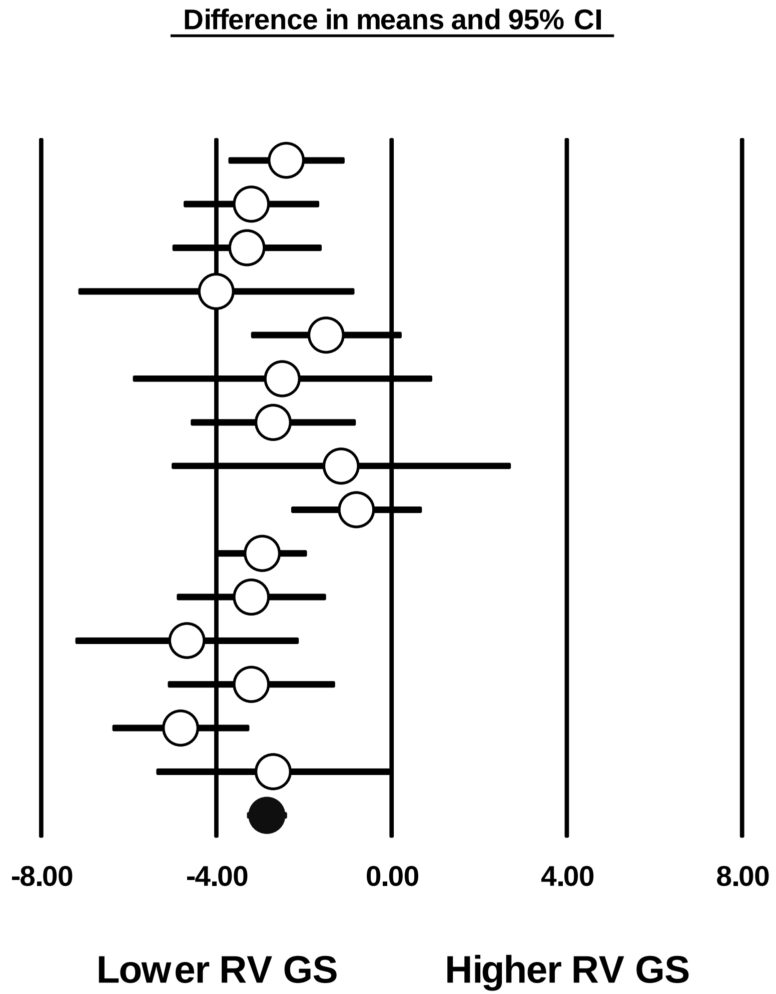

RV FWS, Sclerosis vs Control, Mean Difference, IV, Random, 95% CI

Study name	Difference in means	Standard error	Variance	Lower limit	Upper limit	Z-Value	p-Value
Pigatto et al, 2015	-1.300	0.968	0.937	-3.197	0.597	-1.343	0.179
Tadic et al, 2019	-3.800	0.977	0.954	-5.714	-1.886	-3.891	0.000
Tountas et al, 2019	-2.600	0.752	0.565	-4.073	-1.127	-3.459	0.001
Yiu et al, 2016	-4.000	0.940	0.884	-5.843	-2.157	-4.255	0.000
Zairi et al, 2018 Apical	-7.760	1.758	3.091	-11.206	-4.314	-4.414	0.000
Zairi et al, 2018 Mid	-9.520	1.688	2.850	-12.829	-6.211	-5.639	0.000
Zairi et al, 2018 Basal	-6.800	1.731	2.998	-10.193	-3.407	-3.927	0.000
Norouzi et al, 2022	-3.620	0.907	0.822	-5.397	-1.843	-3.993	0.000
	-4.492	0.794	0.630	-6.048	-2.937	-5.659	0.000

Statistics for each study

Difference in means and 95% CI

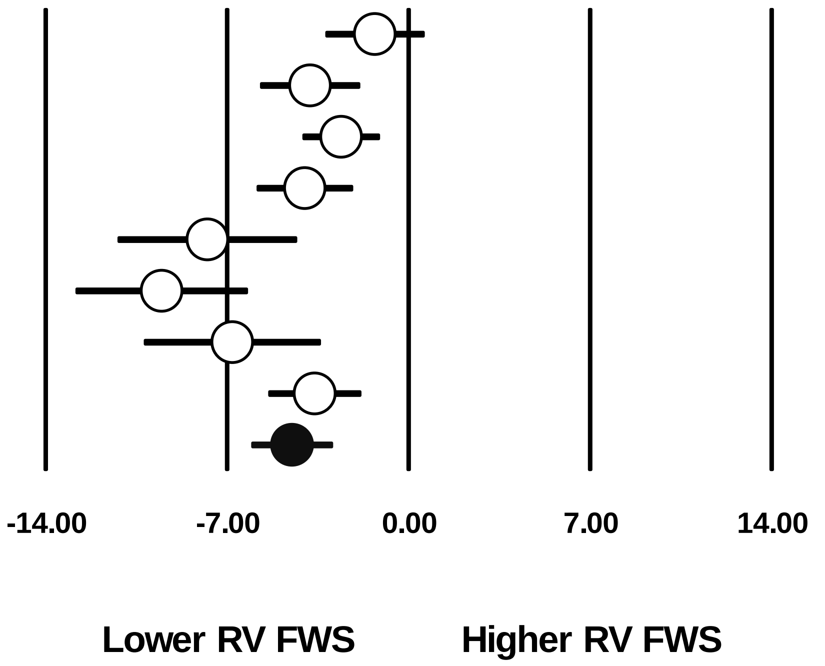

LA Reservoir Strain, Sclerosis vs Control, Mean Difference, IV, Random, 95% CI

Study name	Statistics for each study							Difference in means and 95% CI
	Difference in means	Standard error	Variance	Lower limit	Upper limit	Z-Value	p-Value	
Agoston et al, 2014	-3.700	1.340	1.795	-6.326	-1.074	-2.761	0.006	
Atas et al, 2016	-17.100	2.557	6.537	-22.111	-12.089	-6.688	0.000	
Porpaczy et al, 2018	-10.700	1.733	3.004	-14.097	-7.303	-6.173	0.000	
Tadic et al, 2017	-6.000	0.921	0.848	-7.805	-4.195	-6.514	0.000	
Tigen et al, 2014	-4.700	3.324	11.052	-11.216	1.816	-1.414	0.157	
Sharifkazemi et al, 2022	-9.000	3.998	15.987	-16.837	-1.163	-2.251	0.024	
	-8.317	1.814	3.291	-11.873	-4.761	-4.584	0.000	

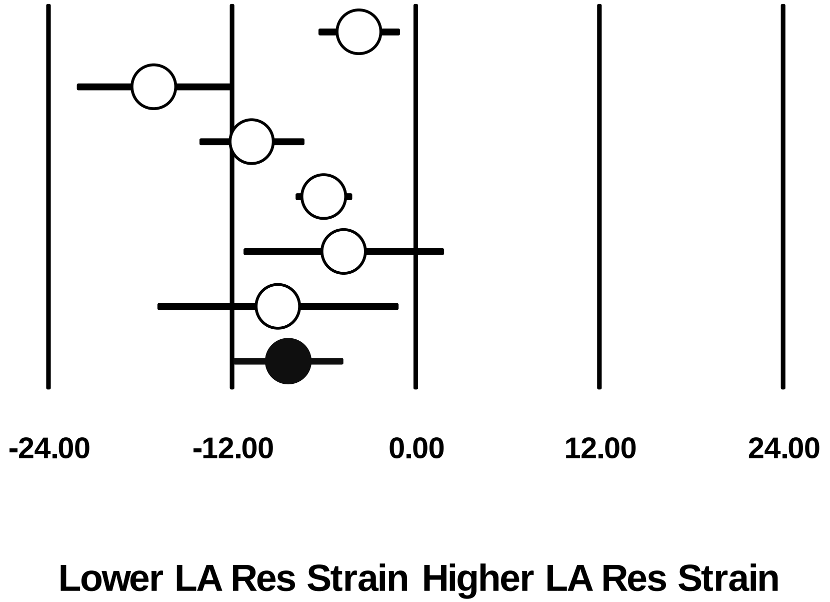

LA Booster Strain, Sclerosis vs Control, Mean Difference, IV, Random, 95% CI

Study name	Difference in means	Standard error	Variance	Lower limit	Upper limit	Z-Value	p-Value
Agoston et al, 2014	-0.700	0.556	0.310	-1.790	0.390	-1.258	0.208
Atas et al, 2016	-0.300	0.253	0.064	-0.796	0.196	-1.185	0.236
Porpaczy et al, 2018	-6.000	0.945	0.892	-7.852	-4.148	-6.351	0.000
Tadic et al, 2017	2.000	0.831	0.690	0.372	3.628	2.408	0.016
	-1.163	1.096	1.201	-3.311	0.985	-1.061	0.289

Difference in means and 95% CI

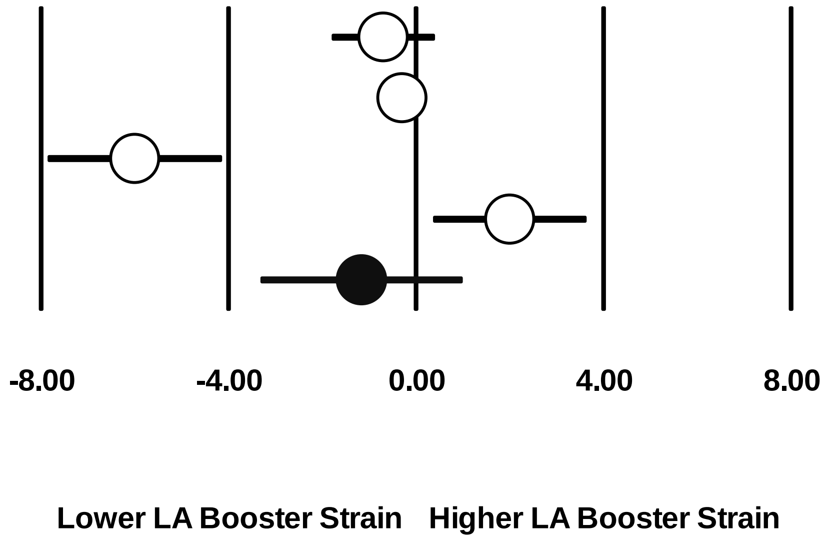

LA Conduit Strain, Sclerosis vs Control, Mean Difference, IV, Random, 95% CI

Study name	Statistics for each study							Difference in means and 95% CI
	Difference in means	Standard error	Variance	Lower limit	Upper limit	Z-Value	p-Value	
Agoston et al, 2014	-3.000	1.325	1.756	-5.597	-0.403	-2.264	0.024	
Atas et al, 2016	-3.100	1.500	2.249	-6.039	-0.161	-2.067	0.039	
Porpaczy et al, 2018	-4.800	1.135	1.288	-7.024	-2.576	-4.229	0.000	
Tigen et al, 2014	1.000	2.003	4.013	-2.926	4.926	0.499	0.618	
	-2.875	1.057	1.117	-4.947	-0.804	-2.720	0.007	

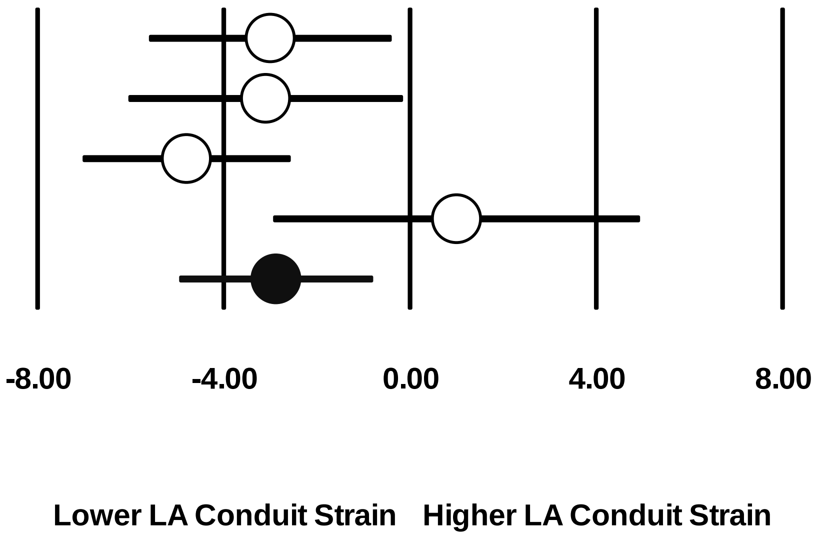

Supplement: Supplementary file 5 — Supplementary Material 5. [file 44156_2025_81_MOESM5_ESM.pdf]
